# Assessment of Awake Prone Positioning in Hospitalized Adults With COVID-19: A Nonrandomized Controlled Trial

**DOI:** 10.1001/jamainternmed.2022.1070

**Published:** 2022-04-18

**Authors:** Edward Tang Qian, Cheryl L. Gatto, Olga Amusina, Mary Lynn Dear, William Hiser, Reagan Buie, Sunil Kripalani, Frank E. Harrell, Robert E. Freundlich, Yue Gao, Wu Gong, Cassandra Hennessy, Jillann Grooms, Megan Mattingly, Shashi K. Bellam, Jessica Burke, Arwa Zakaria, Eduard E. Vasilevskis, Frederic T. Billings, Jill M. Pulley, Gordon R. Bernard, Christopher J. Lindsell, Todd W. Rice

**Affiliations:** 1 Division of Pulmonary, Allergy, and Critical Care Medicine, Department of Medicine, Vanderbilt University Medical Center, Nashville, Tennessee; 2 Vanderbilt Institute for Clinical and Translational Research, Vanderbilt University Medical Center, Nashville, Tennessee; 3 Department of Biostatistics, Vanderbilt University School of Medicine, Nashville, Tennessee; 4 Critical Care Services, NorthShore University HealthSystem, Evanston, Illinois; 5 Department of Biobehavioral Nursing Science, University of Illinois, Chicago, College of Nursing, Chicago; 6 Section of Hospital Medicine, Division of General Internal Medicine and Public Health, Department of Medicine, Vanderbilt University Medical Center, Nashville, Tennessee; 7 Department of Biomedical Informatics, Vanderbilt University Medical Center, Nashville, Tennessee; 8 Division of Anesthesiology Critical Care Medicine, Department of Anesthesiology, Vanderbilt University Medical Center, Nashville, Tennessee; 9 School of Nursing and Health Sciences, North Park University, Chicago, Illinois; 10 Division of Pulmonary and Critical Care, Department of Medicine, NorthShore University HealthSystem, Evanston, Illinois

## Abstract

**Importance:**

Awake prone positioning may improve hypoxemia among patients with COVID-19, but whether it is associated with improved clinical outcomes remains unknown.

**Objective:**

To determine whether the recommendation of awake prone positioning is associated with improved outcomes among patients with COVID-19–related hypoxemia who have not received mechanical ventilation.

**Design, Setting, and Participants:**

This pragmatic nonrandomized controlled trial was conducted at 2 academic medical centers (Vanderbilt University Medical Center and NorthShore University HealthSystem) during the COVID-19 pandemic. A total of 501 adult patients with COVID-19–associated hypoxemia who had not received mechanical ventilation were enrolled from May 13 to December 11, 2020.

**Interventions:**

Patients were assigned 1:1 to receive either the practitioner-recommended awake prone positioning intervention (intervention group) or usual care (usual care group).

**Main Outcomes and Measures:**

Primary outcome analyses were performed using a bayesian proportional odds model with covariate adjustment for clinical severity ranking based on the World Health Organization ordinal outcome scale, which was modified to highlight the worst level of hypoxemia on study day 5.

**Results:**

A total of 501 patients (mean [SD] age, 61.0 [15.3] years; 284 [56.7%] were male; and most [417 (83.2%)] were self-reported non-Hispanic or non-Latinx) were included. Baseline severity was comparable between the intervention vs usual care groups, with 170 patients (65.9%) vs 162 patients (66.7%) receiving oxygen via standard low-flow nasal cannula, 71 patients (27.5%) vs 62 patients (25.5%) receiving oxygen via high-flow nasal cannula, and 16 patients (6.2%) vs 19 patients (7.8%) receiving noninvasive positive-pressure ventilation. Nursing observations estimated that patients in the intervention group spent a median of 4.2 hours (IQR, 1.8-6.7 hours) in the prone position per day compared with 0 hours (IQR, 0-0.7 hours) per day in the usual care group. On study day 5, the bayesian posterior probability of the intervention group having worse outcomes than the usual care group on the modified World Health Organization ordinal outcome scale was 0.998 (posterior median adjusted odds ratio [aOR], 1.63; 95% credibility interval [CrI], 1.16-2.31). However, on study days 14 and 28, the posterior probabilities of harm were 0.874 (aOR, 1.29; 95% CrI, 0.84-1.99) and 0.673 (aOR, 1.12; 95% CrI, 0.67-1.86), respectively. Exploratory outcomes (progression to mechanical ventilation, length of stay, and 28-day mortality) did not differ between groups.

**Conclusions and Relevance:**

In this nonrandomized controlled trial, prone positioning offered no observed clinical benefit among patients with COVID-19–associated hypoxemia who had not received mechanical ventilation. Moreover, there was substantial evidence of worsened clinical outcomes at study day 5 among patients recommended to receive the awake prone positioning intervention, suggesting potential harm.

**Trial Registration:**

ClinicalTrials.gov Identifier: NCT04359797

## Key Points

**Question:**

Is prone positioning associated with improved outcomes among patients with COVID-19 and hypoxemia requiring supplemental oxygen but not yet receiving mechanical ventilation?

**Findings:**

In this nonrandomized controlled trial including 501 patients with COVID-19 and hypoxemia, the odds of having a worse outcome on study day 5 based on a modified World Health Organization ordinal scale was higher among patients receiving the awake prone positioning intervention.

**Meaning:**

This study’s findings suggest that routine recommendation for awake prone positioning among patients with COVID-19–related hypoxemia who require supplemental oxygen but not mechanical ventilation is not beneficial.

## Teaser

This nonrandomized controlled trial uses a modified World Health Organization ordinal scale to assess whether awake prone positioning was associated with improved clinical outcomes among patients with COVID-19–related hypoxemia who require supplemental oxygen but have not received invasive mechanical ventilation.

## Introduction

On March 11, 2020, the World Health Organization (WHO) labeled COVID-19 a global pandemic.^1^ Patients with COVID-19 develop acute hypoxemic respiratory failure,^2^ and 71% to 88% of critically ill patients receive invasive mechanical ventilation.^3,4,5^ Numerous pharmacological therapies have been studied to determine best treatment for these patients.^6,7,8,9^

Awake prone positioning among patients not receiving mechanical ventilation represents 1 nonpharmacological therapy included in the consensus guidelines for patients with COVID-19.^10^ Prone positioning has been used in the care of patients with acute respiratory distress syndrome receiving mechanical ventilation since the 1970s.^11^ Changing position from supine to prone may enhance oxygenation by improving ventilation-perfusion matching through recruitment of previously atelectatic dependent alveoli,^12,13^ reducing lung overinflation^14^ and increasing postural clearance of secretions.^15^ Among patients with severe acute respiratory distress syndrome, early prone positioning may confer a mortality benefit.^16,17^ Among patients not receiving mechanical ventilation, prone positioning improves hypoxemia, but improvement is not sustained on return to supine positioning.^18,19,20^ This observation has been replicated in patients with COVID-19.^21,22^

Many retrospective cohort studies have explored the use of awake prone positioning among patients with COVID-19 who were not receiving mechanical ventilation.^23,24,25,26^ Current guidelines recommend awake prone positioning for patients with COVID-19 as a safe and beneficial practice despite a lack of evidence from randomized clinical trials.^10^ Reviews and meta-analyses have reported an association between awake prone positioning and improved clinical outcomes, but rigorous prospective randomized clinical trials are needed to guide clinical management.^27,28,29^ We designed a pragmatic (designed to evaluate the benefits of an intervention in real-life routine practice conditions) nonrandomized controlled trial to assess practitioner-recommended prone positioning and clinical outcomes among patients with COVID-19–related hypoxemia who had not received mechanical ventilation.

## Methods

This nonrandomized controlled trial enrolled patients from 2 academic medical centers, Vanderbilt University Medical Center (primary site; Nashville, Tennessee) and NorthShore University HealthSystem (Evanston, Illinois) between May 13 and December 11, 2020 (trial protocol available in Supplement 1). The clinical trial was presented to each individual institutional review board as posing minimal risk to participants. No evidence at the time of approval suggested superiority of either the prone or supine position for the care of patients with COVID-19, and both would be encountered in usual care. The study was approved by the Vanderbilt University Medical Center with a waiver of informed consent based on a minimal risk determination. The institutional review board of NorthShore University HealthSystem also approved the study with the requirement that all participants provide verbal informed consent to collect their data for research purposes (eMethods 3 in Supplement 2). This study followed the Consolidated Standards of Reporting Trials (CONSORT) reporting guideline.

### Study Design and Population

This pragmatic 2-center nonrandomized controlled trial involved adult patients aged 18 years or older who were hospitalized with acute hypoxemic respiratory failure and documented COVID-19 infection (based on polymerase chain reaction testing) but had not received mechanical ventilation. Acute hypoxemic respiratory failure was defined as the need for supplemental oxygen provided by standard low-flow nasal cannula, high-flow nasal cannula (HFNC), or noninvasive positive-pressure ventilation to maintain an oxygen saturation of 89% or higher.

### Enrollment and Allocation

**Figure 1.**
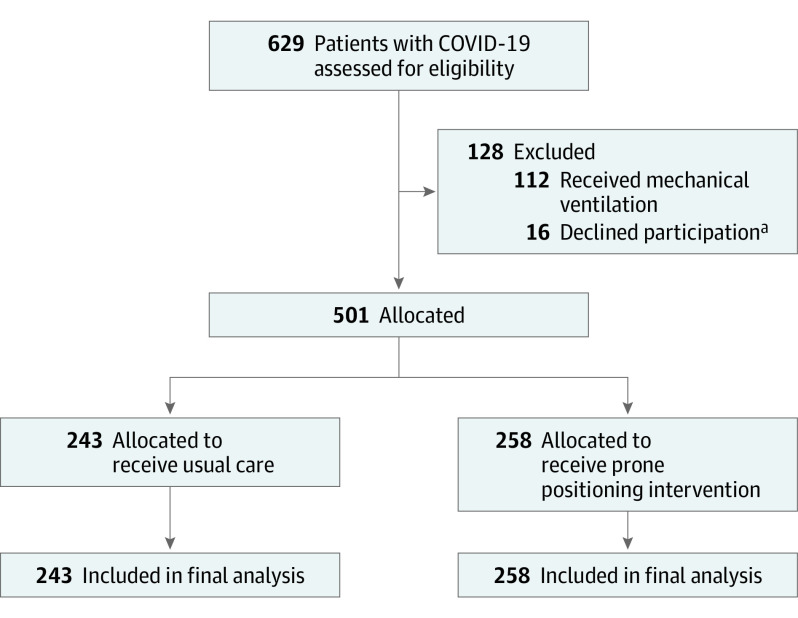
CONSORT Diagram ^a^Patients who declined participation were from NorthShore University HealthSystem.

The medical records of patients with COVID-19 were screened daily for supplemental oxygen use. Patients who received invasive mechanical ventilation previously during the index hospitalization were excluded. Patients not receiving supplemental oxygen at the time of review were marked for follow-up and enrolled if hypoxemia developed during hospitalization. To maximize study inclusivity and encourage diverse recruitment, previous or current receipt of invasive mechanical ventilation represented the only exclusion criterion (Figure 1). We assigned 501 patients to treatment groups based on their medical record numbers; patients with odd numbers were allocated to receive the practitioner-recommended awake prone positioning intervention (intervention group), and patients with even numbers were allocated to receive usual care (usual care group). Usual care was defined as care for COVID-19 without the routine suggestion of prone positioning with usual care for COVID-19 including but not limited to pharmacologic treatment, fluid management, oxygen delivery, and antibiotic administration. Assignment of medical records by hospitals is performed at the time of hospital registration and is not dependent on patient status or specific to the service area of encounter. Patient enrollment and group assignments were stored within the Research Electronic Data Capture system^30^ (eMethods 1 in Supplement 2).

### Intervention

A list of enrolled patients assigned to the intervention group along with practitioner-oriented prone positioning guidelines and patient-oriented prone positioning flyers were distributed to clinicians each morning (eMethods 2 in Supplement 2). Starting on July 10, 2020, after 93 patients were enrolled, an awake prone positioning nursing order was also placed in the patient’s medical record at enrollment. Because the optimal duration of prone positioning was unknown, patients were encouraged by practitioners to use prone positioning as often and consistently as they were able. Practitioners were encouraged to use appropriate clinical judgment for patients who may have had contraindications to prone positioning. Patients assigned to the usual care group were not given any specific direction on positioning but were not prevented from prone positioning if pursued spontaneously or thought to be necessary by the clinical team, which preserved both practitioner and patient autonomy. Given the focus of the intervention on practitioner-recommended prone positioning, practitioners were necessarily unblinded.

### Fidelity Measures

Because patients self-determined the amount of time they spent in a prone position, bedside nurses, who were involved with the recommendation of prone positioning, estimated the time each patient spent in the prone position. Nurses were contacted in person twice daily at the end of each shift to obtain their estimates for the duration of prone positioning for each patient in both groups during their shift. In addition, the team member (E.T.Q.) collecting these estimates noted the patient position at the time of record. Nurses were instructed to consider a patient prone if their position was between 90 degrees (full side) and 180 degrees (face down) in relation to the bed; lying on the side (90 degrees) was not considered a prone position.

### Study Outcomes and Definitions

The primary outcome was the highest level of oxygen support on day 5 after enrollment according to a modified WHO COVID-19 ordinal outcome scale.^31^ To provide more refined discrimination of levels of oxygen support, the WHO ordinal scale was modified to include the maximum fraction of inspired oxygen (FiO_2_) within each ordinal level.^31^ The 8-level scale ranks death as the worst possible outcome followed by extracorporeal membrane oxygenation, invasive mechanical ventilation, noninvasive positive-pressure ventilation, HFNC, standard low-flow nasal cannula or face mask, room air, and discharge to home (best possible outcome). The FiO_2_ level while receiving oxygen via a standard nasal cannula or face mask was estimated as liters of oxygen flow per minute multiplied by 3 and added to 21%. Use of a nonrebreather face mask was the worst ranking within the standard nasal cannula category. The FiO_2_ levels while receiving oxygen via HFNC, noninvasive positive-pressure ventilation, and invasive mechanical ventilation were obtained from the medical record. For patients discharged before study day 5, the last documented outcome status before discharge was used for analysis.

The secondary outcome was the most intensive level of respiratory support used for each patient on each day leading up to study day 5. Exploratory outcomes included length of stay, ventilator-free days, need for invasive mechanical ventilation, maximum FiO_2_ levels on study days 1 through 5, and most severe outcome on the modified WHO ordinal scale on study days 14 and 28. Ventilator-free days were defined as the number of days between the date of extubation and study day 28. If a patient was reintubated, only the date of last extubation was considered. Patients who died before study day 28 were assigned 0 ventilator-free days.

### Statistical Analysis

Although the original statistical analysis plan suggested a frequentist approach, the bayesian statistical design was set a priori (amendment added to the statistical analysis plan is available in Supplement 1). Without previous knowledge about the distribution of the day 5 ordinal outcome available, a single blinded sample size reestimation was conducted to check assumptions about the outcome distribution. Using a frequentist approach and based on the distribution of the ordinal outcome at day 5 among 93 patients, we estimated that enrollment of 500 patients would provide greater than 80% power to detect an odds ratio (OR) of 1.6 with 2-sided α = .05 (using the Whitehead method with the posamsiz function in the Hmisc package for R software, version 4.0.2 [R Foundation for Statistical Computing]).^32,33^ The final statistical analysis plan was subsequently prepared and finalized before data lock and comparative analysis (Supplement 1).

For the primary outcome, a baseline covariate-adjusted bayesian proportional odds ordinal logistic model was used to compare outcomes between study groups, adjusting for age, sex, body mass index (calculated as weight in kilograms divided by height in meters squared), race, ethnicity, Elixhauser comorbidity score, baseline oxygen requirement, enrollment period (May to September 2020 or October to December 2020; based on 2 surges of the pandemic, with enrollment treated the same throughout), and enrollment site. To evaluate the proportional odds assumption and confirm the adjusted odds ratio (aOR) was an appropriate summary measure, the ORs across the full range of cutoff points were estimated. For secondary outcomes, we applied the same approach used for the primary outcome. For exploratory outcomes, binary outcomes were modeled assuming a logit link function, and ordinal and discrete outcomes were modeled using a proportional odds model.

To assess heterogeneity of treatment effect, an interaction term between the baseline patient characteristic of interest and the allocation group was added to the primary model. Missing baseline covariate data were multiply imputed using predictive mean matching, and posterior stacking was used to combine bayesian posterior draws of each separately analyzed completed data set.^34^ Two models were developed: a primary model without an interaction term and a secondary model with an interaction term between treatment and covariate. These models were compared to determine their probability of being the correct model. The differential effect size of prone positioning by baseline level of oxygen support was assessed through an interaction term in a post hoc analysis.

The bayesian ordinal model was fit using the rmsb package for R software, version 4.0.2.^35,36^ Dirichlet priors were set on intercepts, and wide normal priors with a mean (SD) value of 0 (100) were used for all regression coefficients, including treatment. Evidence for efficacy or harm was quantified based on the bayesian posterior probability that the treatment OR would be lower or higher than 1.0. In this analysis, an OR greater than 1.0 was considered a worse outcome. For secondary analyses, all findings were considered to be hypothesis generating. A sensitivity analysis restricted to the primary enrolling site was prespecified. The threshold for statistical significance was 2-tailed *P* = .05.

Due to the unpredictable and dynamic nature of the pandemic, certain analyses were adjusted to accommodate emerging contexts. Therefore, particular covariates, including smoking status, vasopressor use at baseline, and kidney replacement therapy, were not used in the primary analysis. Smoking status was not available for many patients, and vasopressor use and kidney replacement therapy were rare at baseline and lacked sufficient variability to account for in the model. In addition, the model accounted for time epochs after clinical trial completion but before data analysis because patients were enrolled across 2 separate waves of the virus, which could not have been preemptively expected.

## Results

**Figure 2.**
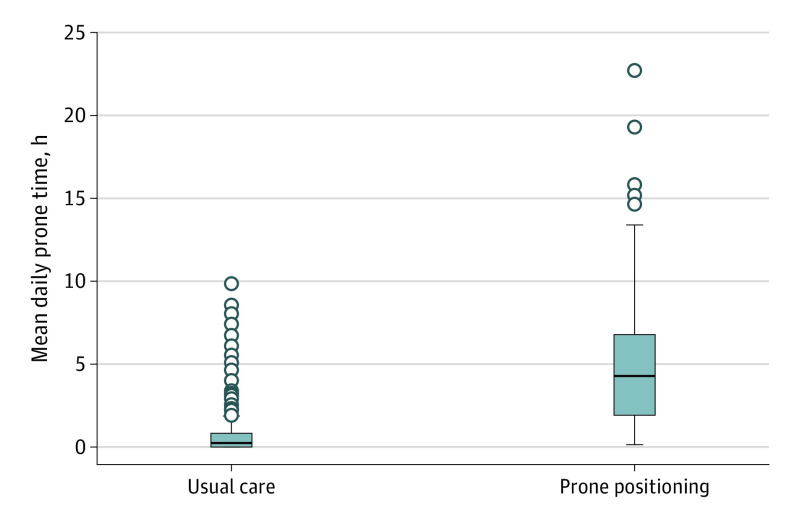
Nursing Estimations of Duration of Prone Positioning The horizontal line inside the boxes represents the median. The whiskers represent distances of 1.5 IQR higher and lower than the third and first quantiles, respectively. The dots represent outliers that are outside of this range.

A total of 501 patients (mean [SD] age, 61.0 [15.3] years; 284 [56.7%] were male; and most [417 (83.2%)] were self-reported non-Hispanic or non-Latinx) were included. Among 258 patients assigned to the intervention group and 243 patients assigned to the usual care group, baseline characteristics were comparable (eg, mean [SD] age, 61.6 [15.4] years vs 60.3 [15.2] years; 146 men [56.6%] vs 138 men [56.8%]; 97 patients [37.6%] vs 91 patients [37.4%] with an Elixhauser comorbidity score <3.0). The level of oxygen delivery at enrollment was also similar between the intervention and usual care groups, with 170 patients (65.9%) vs 162 patients (66.7%) receiving oxygen via standard nasal cannula, 71 patients (27.5%) vs 62 patients (25.5%) receiving oxygen via HFNC, and 16 patients (6.2%) vs 19 patients (7.8%) receiving noninvasive positive-pressure ventilation (Figure 1; Table 1). Nurses estimated that patients in the intervention group spent a median of 4.2 hours (IQR, 1.8-6.7 hours) per day in the prone position over the first 5 days compared with a median of 0 hours (IQR, 0-0.7 hours) per day in the usual care group (Figure 2; eFigure 1 in Supplement 2).

**Table 1.** Participant Demographic and Disease Characteristics at Baseline

Characteristic	No. (%)	
	Usual care group	Prone positioning group
No.	243	258
Baseline severity^a^		
Low flow	162 (66.7)	170 (65.9)
High flow	62 (25.5)	71 (27.5)
NIV	19 (7.8)	16 (6.2)
Unknown or missing	0	1 (0.4)
Age, mean (SD), y	60.3 (15.2)	61.6 (15.4)
Sex		
Female	105 (43.2)	112 (43.4)
Male	138 (56.8)	146 (56.6)
Race^b^		
American Indian or Alaska Native	0	1 (0.4)
Asian	6 (2.5)	8 (3.1)
Black or African American	43 (17.7)	56 (21.7)
White	162 (66.7)	154 (59.7)
Other^c^	26 (10.7)	30 (11.6)
Unknown	6 (2.5)	9 (3.5)
Ethnicity^b^		
Hispanic or Latinx	33 (13.6)	33 (12.8)
Non-Hispanic or non-Latinx	204 (84.0)	213 (82.6)
Unknown	6 (2.5)	12 (4.7)
BMI, mean (SD)	31.1 (7.7)	32.8 (9.1)
Elixhauser comorbidity score		
Median (IQR)^d^	2.0 (−2.0 to 8.0)	3.0 (−1.0 to 7.0)
Score group		
<3	91 (37.4)	97 (37.6)
≥3	90 (37.0)	103 (39.9)
Unknown or missing	62 (25.5)	58 (22.5)
Clinical measurements		
Creatinine, mean (SD), mg/dL^e^	1.56 (2.09)	1.29 (1.26)
WBCs, mean (SD), ×10^3^/μL^f^	9.0 (9.7)	8.5 (4.9)
Platelets, mean (SD), ×10^3^/μL^g^	234 (92)	219 (86)
ALT, mean (SD), U/L^h^	47.5 (81.3)	38.3 (33.9)
AST, mean (SD), U/L^i^	53.9 (57.7)	51.0 (40.9)
CRP, mean (SD), mg/L^j^	124 (91)	125 (94)
Treatment		
Glucocorticoid medication	184 (75.7)	215 (83.3)
Remdesivir	108 (44.4)	132 (51.2)
Anti–IL-6 therapy	1 (0.4)	0
Time from eligibility to enrollment, mean (SD), h	10.98 (7.18)	12.34 (11.40)

Abbreviations: ALT, alanine aminotransferase; AST, aspartate aminotransferase; BMI, body mass index (calculated as weight in kilograms divided by height in meters squared); CRP, C-reactive protein; IL, interleukin; NIV, noninvasive ventilation; WBC, white blood cell.

SI conversion factors: To convert creatinine to micromoles per liter, multiply by 88.4; white blood cell count to 10^9 ^per liter, multiply by 0.001; platelet count to 10^9 ^per liter, multiply by 1.0; ALT and AST to microkatals per liter, multiply by 0.0167.

^a^ Patients were enrolled only if they were receiving noninvasive supplemental oxygen.

^b^ Race and ethnicity were self-reported.

^c^ Races in the *other* category were not specified in the electronic medical record.

^d^ Patient comorbidities were categorized based on diagnostic codes from the *International Classification of Diseases, Tenth Revision*, which can be used to estimate in-hospital mortality.

^e^ Based on 457 participants.

^f^ Based on 456 participants.

^g^ Based on 457 participants.

^h^ Based on 436 participants.

^i^ Based on 436 participants.

^j^ Based on 385 participants.

**Figure 3.**
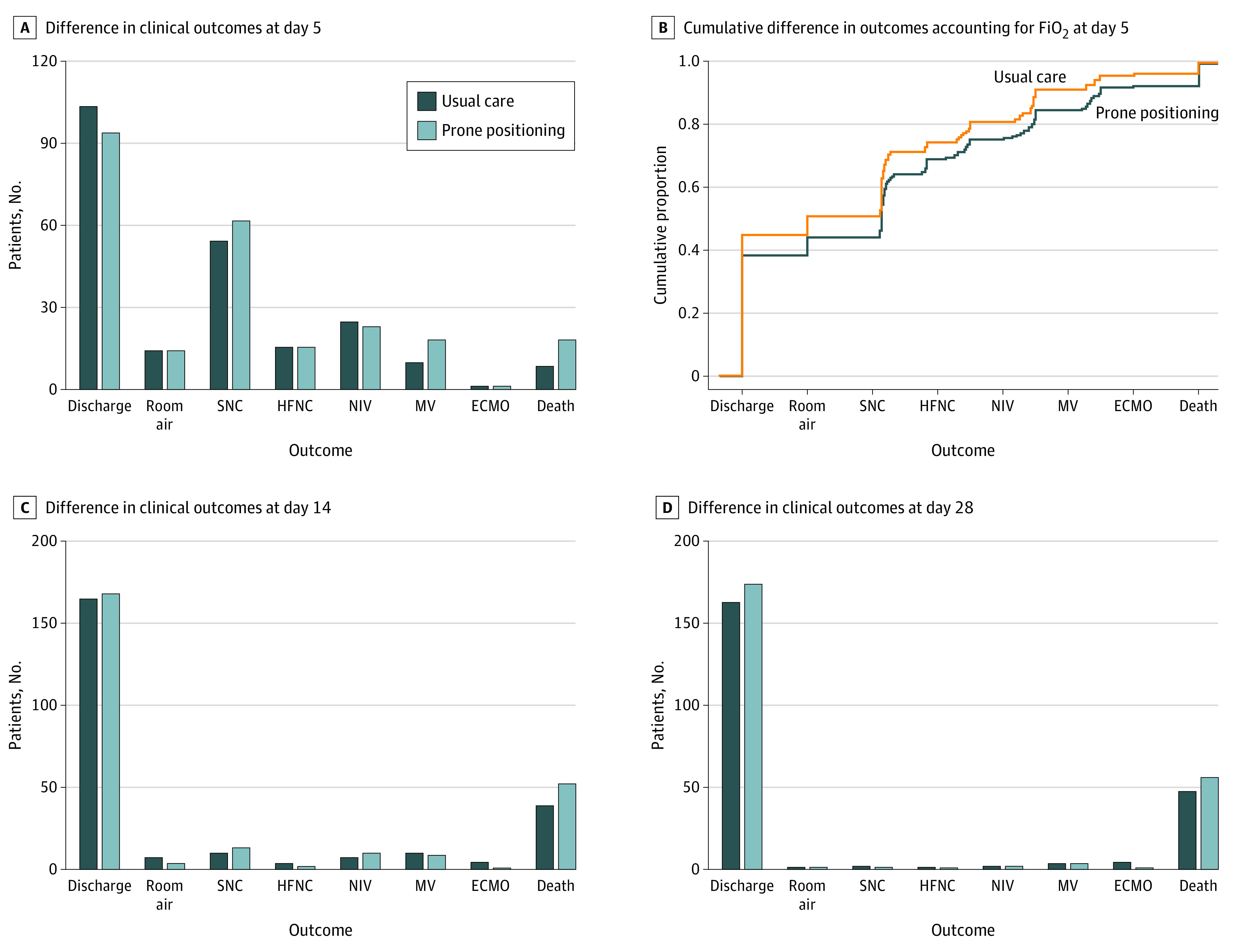
World Health Organization Ordinal Scale Clinical Outcomes at Study Days 5, 14, and 28 A, Differences in clinical outcomes on study day 5. B, Differences in FiO_2_ delivered within each applicable ordinal level by group on day 5. C, Two participants (1 from the usual care group and 1 from the prone positioning group) had missing or unknown data on study day 14. D, Data shown for study day 28 reflect 461 patients from Vanderbilt University Medical Center only. ECMO indicates extracorporeal membrane oxygenation; FiO_2_, fraction of inspired oxygen; HFNC, high-flow nasal cannula; MV, mechanical ventilation; NIV, noninvasive ventilation; and SNC, standard nasal cannula.

The WHO Ordinal Scale Clinical Outcomes at study days 5, 14, and 28 are depicted in Figure 3. On study day 5, the intervention group had a posterior probability of 0.998 for having a worse outcome on the modified WHO COVID-19 ordinal scale (posterior median aOR, 1.63; 95% high-density credibility interval [CrI], 1.16-2.31, in which aOR≥1.0 indicated the intervention had shifted the ordinal outcome toward worse rankings) (Table 2).

**Table 2.** Outcome-Associated Odds Ratios and Additional Exploratory Outcomes^a^

Outcome	Outcome measures				Additional exploratory measures, mean (SD)	
	OR (95% CrI)	Posterior probability^b^	aOR (95% CrI)	Posterior probability^b^	Prone positioning group	Usual care group
**Primary analysis**						
Worst outcome in prone positioning group						
Day 5	1.37 (1.00-1.88)	0.975	1.63 (1.16-2.31)	0.998	NA	NA
**Secondary analysis**						
Worst outcome in prone positioning group						
Day 0	1.01 (0.68-1.50)	0.520	1.02 (0.63-1.63)	0.527	NA	NA
Day 1	1.16 (0.83-1.62)	0.812	1.16 (0.81-1.67)	0.792	NA	NA
Day 2	1.07 (0.78-1.47)	0.666	1.06 (0.74-1.46)	0.625	NA	NA
Day 3	1.17 (0.85-1.59)	0.836	1.22 (0.88-1.70)	0.879	NA	NA
Day 4	1.28 (0.94-1.77)	0.939	1.39 (0.99-1.94)	0.972	NA	NA
**Exploratory analysis**						
Worst outcome in prone positioning group						
Day 14	1.16 (0.81-1.67)	0.783	1.29 (0.84-1.99)	0.874	NA	NA
Day 28^c^	1.04 (0.67-1.56)	0.568	1.12 (0.67-1.86)	0.673	NA	NA
**Additional exploratory analysis**						
Maximum FiO_2_^d^						
Day 1	NA	NA	NA	NA	45.32 (29.08)	40.36 (27.10)
Day 2	NA	NA	NA	NA	43.96 (30.85)	40.28 (28.20)
Day 3	NA	NA	NA	NA	43.60 (32.09)	39.31 (29.78)
Day 4	NA	NA	NA	NA	42.66 (32.52)	37.82 (30.24)
Day 5	NA	NA	NA	NA	40.59 (31.98)	37.10 (30.97)
Length of stay, d						
Hospital	NA	NA	NA	NA	8.20 (10.16)	9.18 (13.09)
ICU	NA	NA	NA	NA	3.36 (8.11)	3.81 (10.63)
Ever intubated during study, No./total No. (%)	NA	NA	NA	NA	31/258 (12.0)	30/243 (12.3)
28-d Hospital mortality, No./total No. (%)^c^	NA	NA	NA	NA	56/239 (23.4)	47/222 (21.2)
Ventilator-free days to day 28^c^^,^^e^						
Mean (SD)	NA	NA	NA	NA	26.95 (3.81)	26.50 (4.75)
Median (IQR)	NA	NA	NA	NA	28.0 (28.0-28.0)	28.0 (28.0-28.0)

Abbreviations: aOR, adjusted odds ratio; CrI, credibility interval; FiO_2_, fraction of inspired oxygen; ICU, intensive care unit; NA, not applicable; OR, odds ratio.

^a^ Adjusted and unadjusted ORs describing the effect of prone positioning on outcomes with additional exploratory outcomes.

^b^ Probability that awake prone positioning had a worse modified World Health Organization ordinal outcome scale status than usual care.

^c^ Includes patients from Vanderbilt University Medical Center (primary site) only.

^d^ Maximum FiO_2_ is the highest fraction of FiO_2_ on the highest level of oxygen support (nasal cannula, high-flow nasal cannula, or noninvasive ventilation) on the specified day. The FiO_2_ for high-flow nasal cannula, noninvasive positive pressure ventilation, and mechanical ventilation were obtained from the medical record. The FiO_2_ for low-flow oxygen was calculated as 3% multiplied by liters of oxygen flow per minute plus 21%.

^e^ Ventilator-free days were calculated as the number of days to day 28 since the date of the first full day after the final extubation event. Patients who died before day 28 were assigned a value of 0 regardless of whether they had any days off of mechanical ventilation.

Using a model without covariate adjustment, the median estimated unadjusted OR was 1.37 (95% CrI, 1.00-1.88), with a posterior probability of 0.975 for an OR of 1.0 or greater (Table 2). Among patients enrolled at the primary site (Vanderbilt University Medical Center), the estimated aOR was 1.52 (95% CrI, 1.08-2.17), with a posterior probability of 0.990 for worse ordinal outcomes among the intervention group at study day 5 (eResults 1 in Supplement 2).

The intervention did not demonstrate any significant effect size of heterogeneity with regard to baseline patient characteristics. All interaction models, including enrollment period, baseline severity, age, sex, race, ethnicity, body mass index, Elixhauser comorbidity score, and enrollment site, had lower probability of being the true model than the primary model; the model weights for the models without interaction terms all exceeded 0.5 (eFigures 2-8 in Supplement 2). The finding of a higher level of oxygen support in the intervention group was consistent across all ordinal levels of oxygen support at baseline (standard low-flow nasal cannula, HFNC, and noninvasive positive-pressure ventilation). The model without the interaction term was 4 times more likely to be true than the model with the interaction term (0.81 vs 0.19) (eFigure 3 in Supplement 2).

On the day of enrollment, there was no evidence that the worst WHO ordinal scale clinical status was different between the 2 study groups (eResults 2 and eTable 1 in Supplement 2). Assessed daily, the posterior probability between the intervention and usual care groups did not exceed 0.950 until study day 4, when the posterior median aOR was 1.39 (95% CrI, 0.99-1.94), and the posterior probability of an aOR of 1.0 or greater was 0.972 (Table 2; eResults 3-6 and eTables 2-5 in Supplement 2).

On study day 14, the posterior median aOR for the modified WHO COVID-19 ordinal scale was 1.29 (95% CrI, 0.84-1.99), and the posterior probability of an aOR of 1.0 or greater was 0.874 (Table 2). At study day 28, the aOR for the posterior median was 1.12 (95% CrI, 0.67-1.86), and the posterior probability of an aOR of 1.0 or greater was 0.673 (Table 2). Within 5 days of enrollment, 19 patients (7.4%) in the intervention group and 9 patients (3.7%) in the usual care group died (eTable 6 in Supplement 2). At study day 14, 52 patients (20.2%) in the intervention group and 38 patients (15.6%) in the usual care group died (eTable 7 and eTable 8 in Supplement 2). At study day 28, for which data were only available for 461 patients from the primary enrollment site, 56 of 239 patients (23.4%) in the intervention group and 47 of 222 patients (21.2%) in the usual care group died (eTable 9 and eTable 10 in Supplement 2). There was no evidence of a difference in either ventilator-free days (median, 28.0 days [IQR, 28.0-28.0 days] in both groups) or the number of patients ever progressing to mechanical ventilation during the study (30 patients [12.3%] in the usual care group vs 31 patients [12.0%] in the intervention group) (Table 2).

A higher maximum FiO_2_ was delivered in the intervention group compared with the usual care group on study days 1 through 5 (eg, day 1: mean [SD], 45.32 [29.08] vs 40.36 [27.10]; day 5: mean [SD], 40.59 [31.98] vs 37.10 [30.97]) (Table 2). When assessing the WHO ordinal scale on each day, the odds of being in a worse outcome rank for oxygen delivery in the intervention group increased from study days 2 through 5 (eg, day 2: aOR, 1.06 [95% CrI, 0.74-1.46; *P* = .38]; day 3: aOR, 1.22 [95% CrI, 0.88-1.70; *P* = .12]; day 4: aOR, 1.39 [95% CrI, 0.99-1.94; *P* = .03]).

## Discussion

In this pragmatic nonrandomized controlled trial of patients with hypoxemia and COVID-19 who had not received mechanical ventilation, a practitioner recommendation of awake prone positioning provided no observed clinical benefit, and it was highly probable that prone positioning worsened patient outcomes at study day 5. Many health care professionals have adopted the recommendation of prone positioning to improve oxygenation and potentially prevent the need for invasive mechanical ventilation, with the hope of increasing survival among patients with COVID-19. However, while awake prone positioning may improve refractory hypoxemia, the data from this study suggest that a universal recommendation of awake prone positioning for the care of patients with COVID-19–associated hypoxemia who are not yet receiving mechanical ventilation may worsen clinical outcomes and cause harm. There are several potential explanations for these findings. A higher proportion of patients were discharged to home within 5 days in the usual care group compared with the intervention group. For patients receiving oxygen via standard nasal cannula, prone positioning may have had little or no relevance to clinical status but prompted practitioners to determine that the patient was less ready for discharge to home because the patient was receiving an intervention to maintain oxygen saturation. This explanation would not account for the higher probability of worse clinical outcomes on study day 5 that was observed in the intervention group.

Prone positioning early in the disease process may accelerate the progression of lung damage, which is supported by the increasing odds of having a higher ranking of oxygen support from study days 2 to 5 observed in the intervention group. Adoption of early prone positioning may improve oxygenation but obscure the natural progression of disease, causing practitioners to potentially delay life-saving therapies or diagnostic testing.

The act of changing position may dislodge oxygen support, leading to desaturation events and potentially prompting urgent intubation and worse outcomes. Although these events were not systematically collected, the steadily worsening oxygenation observed in the intervention group does not support this potential explanation. Although similar numbers of patients in both groups progressed to mechanical ventilation over 28 days, more patients in the intervention group received mechanical ventilation during the first 5 days, suggesting no delay in time to intubation with the use of prone positioning.

A recent meta-trial of 6 randomized clinical trials^37^ including selected patients with COVID-19 receiving oxygen via HFNC and excluding those with relative contraindications for prone positioning reported a reduction in intubation rates at day 28, with a duration of nurse-reported prone positioning that was similar to the duration observed in our study. Intubation rates in the meta-trial were higher than ours, which may reflect differences in intubation practices, particularly given the short time from enrollment to intubation reported in the meta-trial. Despite the decrease in intubation rates, mortality at day 28 of the meta-trial was similar between the groups and similar to mortality in our study. Our clinical trial did not exclude patients with relative contraindications to prone positioning and enrolled patients receiving all levels of noninvasive oxygen support rather than enrolling only those receiving oxygen via HFNC. However, post hoc analyses suggested our results were consistent across all levels of baseline oxygen support. Recent data have revealed that awake patients do not tolerate prone positioning well.^37,38,39^ The outcomes associated with spending more daily time in the prone position remain unknown. However, patients in the intervention group had worse clinical outcomes despite spending relatively short amounts of time in the prone position each day.

### Strengths and Limitations

This study has several strengths. First, the study was a large pragmatic nonrandomized controlled trial of awake prone positioning and clinical outcomes among patients with hypoxemia with COVID-19. Second, the finding of worse outcomes among patients allocated to the awake prone positioning group was consistent across many post hoc sensitivity analyses. Third, this pragmatic clinical trial included patients who may have had relative contraindications to prone positioning. Although this inclusion might have produced bias toward the null, it increased the overall generalizability of the results. Fourth, enrollment was not limited to specific types of noninvasive oxygen delivery, allowing for inclusivity of varying disease severities. The results were consistent regardless of baseline level of oxygen support.

This study also has limitations. First, instead of traditional randomization, patients were allocated to treatment groups using medical record numbers. This approach avoided delays in the initiation of the study intervention and facilitated inclusion of sites. The study team and practitioners were not able to change medical record numbers before enrollment, and all consecutive eligible patients were approached for enrollment without the practitioner having the ability to exclude eligible patients. A small percentage of participants who were unaware of clinical trial interventions declined to have their data collected and were subsequently not enrolled in the clinical trial at 1 institution (NorthShore University HealthSystem).

Second, oxygen saturation was not measured before, during, or after prone positioning. The association between prone positioning and oxygen saturation has already been well documented in a number of physiological studies,^18,19,20,21,22^ which allowed us to focus on clinically relevant outcomes. However, in our clinical trial, awake prone positioning did not seem to decrease progression to mechanical ventilation.

Third, although this study involved multiple centers, most patients were enrolled at a single center (Vanderbilt University Medical Center). Due to the urgency of understanding treatments during the pandemic, a decision was made to not delay local enrollment at Vanderbilt University Medical Center while securing institutional review board approval from other potential sites. NorthShore University HealthSystem subsequently began enrollment after receiving regulatory authorization. The treatment effect was similar regardless of the site of enrollment, suggesting generalizability.

Fourth, as the pandemic evolved, the enrolled patient population shifted to requiring higher baseline levels of inspired oxygen. Although there may be a fundamental difference in the outcomes associated with prone positioning among patients with higher disease severity, both the time period and oxygen delivery method at enrollment were accounted for in the adjusted model, with consistent results across all levels of baseline oxygen support. This consistency suggests that the detrimental consequences associated with early awake prone positioning persist despite the severity of illness at presentation.

Fifth, due to the pragmatic nature of this clinical trial, the clinical team, who might have been aware of patient assignment to the prone positioning group, made decisions on the levels of oxygen support to provide and the timing of discharge to home. However, oxygen titration was standardized via protocol by respiratory therapy in the clinical care of these patients, including throughout their clinical trial participation.

Sixth, the intervention comprised practitioner-recommended awake prone positioning, which resulted in patients spending relatively little time overall in the prone position. Nursing estimates were used to assess the duration of prone positioning, which may have produced bias given the unblinded nature of the study. In an attempt to validate these estimates with biometric sensors, data were only collected from a small number of patients in the final weeks of the clinical trial. Subsequent study and analysis are warranted. Seventh, the data in this study do not provide information on the clinical outcomes associated with rescue awake prone positioning among patients with acute hypoxemia and worsening conditions because this practice was also allowed in the usual care group.

## Conclusions

In this nonrandomized controlled trial of patients with COVID-19–associated hypoxemia who did not receive invasive mechanical ventilation, practitioner recommendation for awake prone positioning resulted in no observed clinical benefit and a high probability of worse clinical outcomes on study day 5. These findings suggest that routine preferential use of prone positioning among patients with COVID-19 who require supplemental oxygen but are not receiving invasive mechanical ventilation may not be associated with patient benefits.

## Notes

Supplement 1.
Trial Protocol, Original Statistical Analysis Plan, and Amended Statistical Analysis Plan

Supplement 2. **eMethods 1.** Enrollment, Allocation, and Data Extraction **eMethods 2.** Materials Provided to the Clinical Staff Assisting With the Study **eMethods 3.** Regulatory Considerations **eResults 1.** Worst Status on Day 5, Adjusted **eResults 2.** Worst Status on Day 0 **eResults 3.** Worst Status on Day 1 **eResults 4.** Worst Status on Day 2 **eResults 5.** Worst Status on Day 3 **eResults 6.** Worst Status on Day 4 **eTable 1.** WHO Ordinal Scale at Enrollment (Day 0) **eTable 2.** WHO Ordinal Scale at Day 1 **eTable 3.** WHO Ordinal Scale at Day 2 **eTable 4.** WHO Ordinal Scale at Day 3 **eTable 5.** WHO Ordinal Scale at Day 4 **eTable 6.** Worst Outcome at Day 5 by Assigned Treatment (Table 3 in Statistical Report) **eTable 7.** Worst Outcome on WHO Ordinal Scale at Day 14 by Assigned Treatment (Table 6 in Statistical Report) **eTable 8.** Worst Outcome on Main Ordinal Scale at Day 14 by Assigned Treatment (Table 9 in Statistical Report) **eTable 9.** Worst Outcome on WHO Ordinal Scale at Day 28 by Assigned Treatment (Table 8 in Statistical Report) **eTable 10.** Worst Outcome on Main Ordinal Scale at Day 28 by Assigned Treatment (Table 10 in Statistical Report) **eFigure 1.** Average Daily Prone Time for All Participants With Nursing Data Available by Treatment Assignment (Figure 4 in Statistical Report) **eFigure 2.** Enrollment Time by Treatment Interaction **eFigure 3.** Baseline Severity by Treatment Interaction **eFigure 4.** Age by Treatment Interaction **eFigure 5.** Sex by Treatment Interaction **eFigure 6.** Race by Treatment Interaction **eFigure 7.** Elixhauser Score by Treatment Interaction **eFigure 8.** Site by Treatment Interaction

Supplement 3.
Nonauthor Collaborators

Supplement 4.
Data Sharing Statement
