# Supplementary material for: Assessment of Awake Prone Positioning in Hospitalized Adults With COVID-19: A Nonrandomized Controlled Trial
Source: JAMA Intern Med. 2022 Apr 18;182(6):612–21. doi: 10.1001/jamainternmed.2022.1070 (PMC9016608; doi:10.1001/jamainternmed.2022.1070)
Supplement: Supplement 1. — Trial Protocol, Original Statistical Analysis Plan, and Amended Statistical Analysis Plan [file jamainternmed-e221070-s001.pdf]

# Supplemental Appendix 1 – Study Protocol

## Pragmatic Trial Exploring Impact of Patient Positioning in the Management of Patients Infected with COVID-19: Supine vs. Prone


**Study Co-Principal Investigators:**

Todd Rice, MD, MSc, Associate Professor, Department of Medicine; Director, Medical ICU; Director, Vanderbilt Human Research Protections Program

Edward Qian, MD, Fellow, Department of Medicine

Cheryl Gatto, PhD, PMP, Research Assistant Professor, Department of Biostatistics; Associate Director, Vanderbilt Institute for Clinical and Translational Research
Vanderbilt University Medical Center

**Co-investigators and KSP**

Gordon Bernard, MD, APCCM
Vanderbilt Institute for Clinical and Translational Research, Department of Medicine

Chris Lindsell, PhD
Department of Biostatistics

Sunil Kripalani, MD
Department of Medicine, Center for Clinical Quality and Implementation Research

Frank Harrell, PhD
Department of Biostatistics

Ed Vasilevskis, MD
Department of Medicine, Center for Quality Aging

Josh Billings, MD, MSCI
Department of Anesthesiology

Jessica Burke, MD
Department of Medicine

Arwa Zakaria, DO
Department of Medicine

Rob Freundlich, MD
Department of Anesthesiology, Department of Biomedical Informatics

Reagan Buie, MHA
Vanderbilt Institute of Clinical and Translational Research

Mary Lynn Dear, PhD
Vanderbilt Institute of Clinical and Translational Research

Will Hiser, BA
Vanderbilt Institute of Clinical and Translational Research

**Table of Contents:**

**Study Schema**
**1.0 Background**
**2.0 Rationale and Specific Aims**
**3.0 Inclusion/Exclusion Criteria**
**4.0 Enrollment/Randomization**
**5.0 Study Procedures**
**6.0 Reporting of Adverse Events or Unanticipated Problems**
**7.0 Study Withdrawal/Discontinuation**
**8.0 Statistical Considerations**
**9.0 Privacy/Confidentiality Issues**
**10.0 Follow-up and Record Retention**

## 1. Background

Disease Progression and Timing of Intervention

The intervention described herein focuses on adjustment of patient positioning aimed at improving gas exchange and lung function in patients harboring COVID-19. This intervention will target the inpatient setting generally. This study presents no more than minimal risk beyond that experienced in standard of care. As such, this study will be seeking a waiver of informed consent.

**Intervention timepoints in patients testing positive for COVID-19**

| CLINICAL SETTING | Incubation | Outpatient | Inpatient | ICU |
|---|---|---|---|---|
| SYMPTOMS | No respiratory symptoms | Fever<br>Cough<br>Shortness of breath<br>Increased respiratory rate<br>Chest pain<br>Dyspnea | Pneumonia<br>Pulmonary edema<br>Acute lung injury<br>Hypoxemia | ARDS Mortality |
| CHRONOLOGY | *2-14 days until onset of symptoms* | Days 1-7 | Days 8-14 | Days 15-25 |
| PRAGMATIC OUTCOMES | N/A | Changes in respiratory rate<br>Inpatient admission | WHO COVID ordinal scale<br>Progression to critical illness | Mortality<br>Ventilator-free days |

Scientific/Clinical Rationale for Approach

Since emergence of the novel coronavirus, severe acute respiratory syndrome coronavirus 2 (SARS-CoV-2) now designated coronavirus disease 2019 (COVID-19), one in six affected patients becomes seriously ill (1). The lung appears to be the most susceptible target organ, with a large swath of symptomatic patients struggling with mild upper respiratory tract illness and severe viral pneumonia resulting in respiratory failure. This respiratory failure is often fatal, with one study showing 28% of non-survivors experienced respiratory failure (2). Moreover, 81-97% of patients requiring mechanical ventilation do not survive (3,4).

Like its interaction with Severe Acute Respiratory Syndrome (SARS-CoV), angiotensin converting enzyme 2 (ACE2) is the functional receptor for COVID-19 (5). Viral adherence to host-cell membrane associated ACE2 facilitates the proximity required for viral "spike" mediated genetic material injection. In COVID-19, this spike is 10-20 times more likely to bind ACE2 than SARS (6). ACE2 is expressed in 0.64% of all human lung cells, with 83% of those cells being alveolar epithelial type II (7). In addition, gene ontology enrichment analysis showed that the ACE2-expressing alveolar epithelial type II have high levels of multiple viral process-related genes, including regulatory genes for viral processes, viral life cycle, viral assembly, and viral genome replication, suggesting that the ACE2-expressing alveolar epithelial type II cells facilitate coronaviral replication in the lung (8). Thus, these cells likely serve as a ready reservoir for viral invasion. Perhaps more importantly, alveolar type II cells function to generate and recycle surfactant essential to respiratory activity (9). Surfactant defends against alveolar collapse at low lung volume and protects the lung from injuries/infections caused by inhaled particles and micro-organisms. In COVID-19, if these vital cells are being destroyed, alveolar failure may ensue with severe lung impairment. Thus, interventions that are aimed at improving pressure normalization and alveolar protection may be beneficial in these patients.

Prone positioning (PP) has long been used to combat hypoxemia in acute respiratory distress syndrome (ARDS). Improvements in gas exchange result from improved alveolar ventilation and blood flow redistribution with enhanced perfusion following (10). PP reduces lung over inflation and bolsters alveolar recruitment (11). PP also promotes uniformity of vertical pleural pressure gradients resulting in more uniform alveolar size (12). Considering these physiologic factors together, **we hypothesize PP serves to balance stress and strain within the lungs of COVID-19 positive patients requiring supplemental**

**oxygen, who have yet to be placed on mechanical ventilation during their hospitalization, leading to improved outcomes compared to traditional supine positioning.**

Prior Research Supporting the Positioning Model:
Multiple studies have been conducted that support the use of PP as a proactive treatment to combat hypoxemia in ARDS. Each year, approximately 170,000 people are diagnosed with ARDS, and those diagnosed face mortality rates between 25% and 40% (12,13). The use of PP stretches back to the 1970s, as providers began to search for ways to ameliorate ARDS symptomatology and reduce the then even higher levels of mortality associated with it. Following initial reports that PP significantly improved oxygenation in 70-80% of patients with ARDS, it was adopted as a standard treatment option. Initially, randomized clinical trials struggled to replicate these findings, citing multiple limitations to study enrollment and treatment standardization that made ascertaining conclusive results difficult (13). Only as RCT construction has been refined to accommodate for these limitations have the benefits of PP been more clearly demonstrated.

These beneficial effects have been recently upheld by the landmark PROSEVA study, a multicenter, prospective, randomized, controlled trial, that randomly assigned 466 patients with severe ARDS to undergo prone-positioning sessions of at least 16 hours or to be left in the supine position. Their results demonstrated a significant improvement in both 28- and 90-day mortality rates: "the 28-day mortality was 16.0% in the prone group and 32.8% in the supine group (P<0.001). The hazard ratio for death with prone positioning was 0.39 (95% confidence interval [CI], 0.25 to 0.63). Unadjusted 90-day mortality was 23.6% in the prone group versus 41.0% in the supine group (P<0.001), with a hazard ratio of 0.44 (95% CI, 0.29 to 0.67)" (14).

Per these positive findings, PP has been consistently shown to be an effective mechanism to increase oxygenation in patients with ARDS when implemented under the following conditions: early enlisting of treatment and its consistent maintenance for at least 16 hours per day, and with concurrent use of lung-protective therapies. Translating these findings towards treatment of COVID-19 positive patients seems promising given the similarity of manifested symptoms and complications.

## 2. Rationale and Specific Aims

This study aims to determine if provider-recommended guidance on supine vs. prone positioning of COVID-19 patients requiring supplemental oxygen, who have yet to be placed on mechanical ventilation during their hospitalization, improves outcomes in the inpatient setting. This study will be performed as a pragmatic clinical trial.

We have selected an ordinal primary outcome that reflects increased need and provision of oxygen to maintain patient oxygenation. The scale is similar to the World Health Organization Ordinal Outcome Scale (see Exploratory Outcomes) but provides better discrimination and power to measure the effect of supine vs. proning treatment by ranking patients within each modality of support using the FIO2 he or she receives. This outcome is 1) easy to quantify through documentation of respiratory support modality and the patient's FIO2, 2) patient centric, and 3) effectively measures requirements for oxygenation support.

Data capture will be facilitated by the use of the REDCap Clinical Data Interoperability Services (CDIS) tools. Project team members listed as Key Study Personnel with existing electronic health record (EHR) system access rights will make use of REDCap CDIS tools. These tools are designed to enable transfer of relevant study-related data from the Vanderbilt Research Derivative and/or directly from the EHR into REDCap.

Primary Outcome:
The highest level of support on the 5$^{th}$ day after enrollment according to the following scale adjusted for patient status at enrollment according to the same scale and ranked by mean FIO2 within each category, as appropriate.

- Death
- ECMO
- Mechanical ventilation (ranked by mean FIO2)

- Non-invasive ventilation such as BiPAP (ranked by mean FIO2)
- High flow nasal cannula, e.g. Optiflow, Vapotherm or other similar device (titrated by FiO2%) (ranked by mean FIO2)
- Standard nasal cannula (titrated by L/min up to 15 L/min) or face mask (ranked by mean FIO2)
- Room air

Notes:

- The **mean** value of FIO2 as a weighted average on day 5 is used for outcome assignment ranking.
- FIO2 on standard nasal cannula or face mask is estimated as 21 + 3 x liters per minute (LPM) O2 flow.
- Non-rebreather face mask is highest (worst) rank within the standard nasal cannula or face mask category.
- FIO2 on high flow nasal cannula, e.g. Optiflow, Vapotherm or other similar device is estimated as the %FIO2 on the machine.
- For patients who are discharged or lost to follow up before day 5, the last documented modality and FIO2 will be carried forward.

Secondary Outcomes:
For each day, we will record the most intensive oxygen delivery mode and then, for that highest level of oxygen support device, the max FiO2 while exposed to that device.

Exploratory Outcomes:

- Length of Stay adjusted for the WHO COVID ordinal scale at admittance
- ICU Length of Stay adjusted for the WHO COVID ordinal scale at admittance
- Intubation Measurements and Extubation
  - Intubation yes/no
  - Ventilator-free days (VFDs) out to 28 days
- Oxygen Levels and Respiratory Outcomes
  - Mean FIO2 for each day 1-5 after enrollment
  - Maximum FIO2 for each day 1-5 after enrollment
- Most severe WHO ordinal scale score during inpatient stay
  1. Death
  2. Hospitalized on invasive mechanical ventilation or ECMO
  3. Hospitalized on non-invasive ventilation or high flow nasal cannula
  4. Hospitalized on supplemental oxygen
  5. Hospitalized not on supplemental oxygen
  6. Discharged/not-hospitalized

     Combination of:
     - Not hospitalized with limitation in activity (continued symptoms)
     - Not hospitalized without limitation in activity (no symptoms)
- Modified WHO COVID-19 ordinal scale measurement at day 14
- Incidence of Complications related to Positioning (e.g., accidental line displacement, vomiting, falls)
- Secondary analysis of age by assigned treatment interaction

Fidelity Measurements: Fidelity measurements will include the following when possible:

- Position
- Time to failure (indicating a loss of ability to self-prone, if capturable)
- Nursing estimates of patient prone time as available
- Time spent in prone vs. supine position (approximation based on random sampling)
  - A small subset of participants will be monitored using a wireless, single-use, disposable device adhered to the patient's chest.

**3. Inclusion/Exclusion Criteria**

Inclusion:

- This study will enroll COVID-19 positive patients admitted to VUMC who require supplemental oxygen, who have yet to be placed on mechanical ventilation during their hospitalization. We will use the hospital's operational status definition to identify COVID-positive case status.

Exclusion:

- Per above, patients admitted on mechanical ventilation will be excluded from enrollment.

**4. Enrollment/Randomization**

This study will be performed as a pragmatic controlled clinical trial. Patients fitting inclusion criteria who are admitted to the Vanderbilt University Adult Hospital will be enrolled in this study. Individual patient-level quasi-randomization will be employed. Odd-numbered MRNs will be assigned to the prone positioning and even-numbered MRNs will be assigned to the usual care arm. COVID-19 admissions will be reviewed during the daily COVID huddle, and appropriate study team personnel will facilitate protocol execution, working with the local providers/units.

**5. Study Procedures**

The study procedures in place involve treatment assignment, as well as electronic data capture. Day zero will be defined as the day of enrollment and treatment assignment. Data will originate from the electronic medical record for all patients, as well as from non-invasive patient wearable positioning sensors for a random subset to ensure protocol fidelity. Patients will be assigned to either one of the two comparator arms. Patients and providers will necessarily be unblinded, but outcomes will be analyzed by a blind assessor. Study arm assignment and the corresponding protocol will be discussed at the beginning of each day during the daily COVID-19 huddle. Workflow will include an initial run in period as we expect implementation uptake to take time. Patients enrolled during this ramp-up and optimization period will be allocated to either the prone or usual care group as appropriate, but their data will not be included in the final analysis.

<u>Comparator Arm A:</u> COVID-positive patients who require supplemental oxygen, but are not yet mechanically ventilated, will be encouraged to lay in a completely prone position for as much time as is tolerable during hospitalization. Guidelines to ensure standardization include the following:

- Instructions to care-giving teams on how to frame the prone positioning ask to patients
- Request that clinical team provide reminders to the patient to prone if they are able (as feasible given workloads)
    - o Suggest putting a pillow under pelvis/abdomen and under the lower legs to alleviate lower back strain
    - o Suggest listening to podcast, music, or audiobook
    - o Guidance to manage IV lines, pulse ox and other monitors per nursing unit leadership engagement
- Proning notation could be added to the room white board
- Materials will be provided to promote appropriate positioning (signage for patient beds)

<u>Comparator Arm B:</u> Usual care, patients will remain in their natural choice of position, which is anticipated to favor a supine, semi-recumbent position. Providers will be freely able to alter the protocol at their discretion in light of any patient decompensation.

<u>Fidelity Monitoring randomly assigned within comparator arm A and arm B:</u> A small subset of participants will be monitored using a routine, FDA-approved, wireless, single-use, disposable device adhered to the patient's chest. Upon activation, the sensor immediately begins monitoring the patient's orientation, position, movement and activity. Monitoring capability continues for 21 days. An initial run-in period will inform appropriate sensor assignments for the remainder of the study duration.

**6. Reporting of Adverse Events or Unanticipated Problems**

Participating in the study does not add risk related to treatment. Any risks to patients from participating in the study are limited to the collection of PHI and treatment assignment.

As such, study procedures do not represent a direct risk to participants above what they would incur during usual care. However, we recognize there may be adverse events related to the study. These include unusual clinical occurrences and the loss of privacy. These will be reported according to appropriate timelines. Complications related to clinical care are being captured routinely as outcomes and will be reported to the IRB as appropriate.

The Principal Investigator will be responsible for overseeing the safety of this trial on a daily basis and will be available at any time for questions from providers. The PI will also record adverse events and serious adverse events. Serious adverse events associated with study procedures will be recorded in the study record and reported to the IRB within 10 business days of knowledge. Summary reports will be submitted to the IRB annually and will contain a) the number of adverse events and b) the number of protocol violations and how each was handled.

Regulatory Considerations:

This study is seeking a waiver of informed consent. The study is believed to be minimal risk as patients naturally orient themselves in both supine and prone positioning. There is no current evidence to support that the prone or supine position is superior in alleviating symptom progression in this disease state, demonstrating equipoise between groups. The rights and welfare of participants are not adversely affected by this study, as this is something they would normally encounter in usual care. The addition of a sensor to monitor patients is common practice, and the readout here will document patient positioning choice (15,16). Within the subset to be monitored for protocol fidelity, patient assent will be obtained. Moreover, the request for positioning and device monitoring still allows the patient to maintain autonomy. Providers are also freely able to adjust the positioning of the patient as they see fit. It is impracticable to fully consent patients for this study as this would introduce bias. Making patients aware of prone positioning may encourage supine patients to assume a prone position, when they would not usually do so.

**7. Study Withdrawal/Discontinuation**

Patients will not generally have the option to self-withdraw from the study, however, physicians retain the right to discontinue upon unacceptable patient decompensation.

8. **Statistical Considerations**

The majority of clinical trials in COVID-19 are using the WHO ordinal outcome scale, or some adaptation thereof. This outcome scale differentiates patients based on their need for supplemental oxygen or, if they are not using oxygen, whether they need help or support for activities of daily living. We have adapted this scale for use in this trial. The adaptation, which involves ranking patients within the ordinal categories based on the magnitude of oxygen requirements, adds granularity to the scale and is intended to increase power. We expect prone positioning to have beneficial effects within the ordinal categories, as well as influencing the method of delivering oxygen (e.g. room air, nasal cannula, high flow nasal annual, intubation).

The scale remains ordinal in nature. We will use the approach of last value carried forward for patients who are not observed for the full five days. Patients discharged home will be at the bottom end of the scale (generally room air) while those who die will be scored the worst possible outcome. Patients discharged to long term care facility or similar will have their oxygen needs at transfer noted as their final requirements. We do not expect many patients to fall into this category since the endpoint is being measured relatively close in time to the intervention (5 days), and transfer is likely to occur after much later in the clinical course.

In the absence of any pre-existing information about our ordinal outcome, and rapidly evolving statistical capabilities to analyze these data being led by our group and others around the world, we describe a traditional analytical approach with associated sample size considerations. We recognize that in the coming weeks new methods will greatly improve the statistical efficiency of these trials and allow us to draw conclusions from fewer patients enrolled. Importantly, evolving approaches will be Bayesian in nature, which allows for early looks at the data so that we can understand the measurement properties of our ordinal scale and the impact of prone positioning and adjust as necessary.

We will use a proportional odds model to compare outcomes between study groups. Because it is possible that oxygen needs at day 5 are influenced by baseline oxygen requirements, we will adjust for baseline. This will answer the question: 'for two patients who have the same oxygen requirements at baseline, will the patient who is in the prone positioning arm have lower oxygen requirements at day 5 than the person in the alternative arm'. Our final model will also adjust for age, BMI, smoking status, and baseline comorbidities.

Secondary analyses that involve continuous variables will generally use a proportional odds model with death always coded as 'worst outcome plus one'. This will prevent bias associated with early death in COVID-19, which is known to have a high mortality right among hospitalized patients. For binary outcomes, a logistic model will be used.

*Sample size considerations*
While data on the WHO ordinal scale are beginning to accrue, they are currently insufficient to understand the measurement properties for detailed sample size analyses. This is particularly the case because different institutions have different practices for use of supplemental oxygen, an given the novel nature of the disease it is not yet possible to protocolize this key outcome variable. In addition, we have modified the endpoint to be more granular and thus we expect it to provide greater power for discriminating differences between study groups. However, we are able to make some assumptions about the general distribution of the outcome scale based on the clinical course of patients admitted with oxygen at our institution, and we have powered our study according to that information. A general distribution across the seven-level ordinal scale at day five for patients with no intervention is as follows:

Death: 1%
Intubated: 5%
High flow nasal cannula (or CPAP, BiPAP): 14%
Supplemental oxygen: 30%
Hospitalized not on oxygen:10%
At home with limitations/At home with no limitations: 40%

Our plan is to use a Bayesian approach. However, we have used a frequentist analysis to estimate the likely sample size under several assumptions about effect size. If we assume that we reduce the need for oxygen and thus are able to discharge an additional 20% of participants by day 5, then 150 patients per group will have over 90% power to detect this difference. If we reduce the need for oxygen among a similar proportion of patients but only discharge an additional 10%, then 175 patients per group will have over 80% power to detect the difference. Given these estimates, we will plan to enroll 250 participants per study arm. When we have accrued 25 patients per arm, we will use the information to revise our sample size because we will then have enough information on which to base a Bayesian design.

Following the interim analysis, the power calculation indicates that N=500 total will be needed to detect an OR of 1.6 with 80% power for the modified WHO ordinal scale at day 5. Due to concern over some missing data, potential crossover between arms, and to allow for measurements of body position sensors that are just now in use, an additional 10% of the sample size will be enrolled for a planned total of 550 patients enrolled at all sites.

## 9. Privacy/Confidentiality Issues

At no time during the course of this study, its analysis, or its publication will patient identities be revealed in any manner. The minimum necessary data containing patient or provider identities will be collected. As quickly as feasible, all data collected will be uploaded into a password-protected computerized database maintained within a secure, web-based application for building and managing online databases (REDCap), or stored on secure servers with user-level access control. All patients will be assigned a unique study number for use in the computerized database. At the time of publication all identifiers will be removed.

**10. Follow-up and Record Retention**

Given the adaptive nature of this trial, execution of this study will continue until a statistically significant result has been reached. Thus, we anticipate a study duration of no more than 12 months. For each participant, the study will commence at enrollment and study intervention will last until hospital discharge or in-hospital death. Patient clinical outcomes will be collected up until hospital discharge with final status for certain outcomes assessed with patient follow-up as needed depending on disposition. Identified data in the secure database will be stored for an indefinite period of time to allow for subsequent data analysis and future reference. However, all patients will be assigned a unique study number for use in the computerized database. At the time of publication, all identifiers will be removed.

**References**


1. Q&A on coronaviruses (COVID-19) [Internet]. [cited 2020 Mar 24];Available from: https://www.who.int/news-room/q-a-detail/q-a-coronaviruses
2. Zhou F, Yu T, Du R, et al. Clinical course and risk factors for mortality of adult inpatients with COVID-19 in Wuhan, China: a retrospective cohort study. The Lancet [Internet] 2020 [cited 2020 Mar 24];0. Available from: https://www.thelancet.com/journals/lancet/article/PIIS0140-6736(20)30566-3/abstract
3. Yang X, Yu Y, Xu J, et al. Clinical course and outcomes of critically ill patients with SARS-CoV-2 pneumonia in Wuhan, China: a single-centered, retrospective, observational study. The Lancet Respiratory Medicine [Internet] 2020 [cited 2020 Mar 24];0. Available from: https://www.thelancet.com/journals/lanres/article/PIIS2213-2600(20)30079-5/abstract
4. Feb 24 SS| NR| CN|, 2020. Study of 72,000 COVID-19 patients finds 2.3% death rate [Internet]. CIDRAP. [cited 2020 Mar 24];Available from: http://www.cidrap.umn.edu/news-perspective/2020/02/study-72000-covid-19-patients-finds-23-death-rate
5. Zhou P, Yang X-L, Wang X-G, et al. A pneumonia outbreak associated with a new coronavirus of probable bat origin. Nature 2020;579:270–3.
6. Wrapp D, Wang N, Corbett KS, et al. Cryo-EM structure of the 2019-nCoV spike in the prefusion conformation. Science 2020;367:1260–3.
7. Zhao Y, Zhao Z, Wang Y, Zhou Y, Ma Y, Zuo W. Single-cell RNA expression profiling of ACE2, the putative receptor of Wuhan 2019-nCov. bioRxiv 2020;2020.01.26.919985.
8. Zhang H, Penninger JM, Li Y, Zhong N, Slutsky AS. Angiotensin-converting enzyme 2 (ACE2) as a SARS-CoV-2 receptor: molecular mechanisms and potential therapeutic target. Intensive Care Med [Internet] 2020 [cited 2020 Mar 24];Available from: https://doi.org/10.1007/s00134-020-05985-9
9. Andreeva AV, Kutuzov MA, Voyno-Yasenetskaya TA. Regulation of surfactant secretion in alveolar type II cells. Am J Physiol Lung Cell Mol Physiol 2007;293:L259-271.
10. Johnson NJ, Luks AM, Glenny RW. Gas Exchange in the Prone Posture. Respir Care 2017;62:1097–110.
11. Galiatsou E, Kostanti E, Svarna E, et al. Prone position augments recruitment and prevents alveolar overinflation in acute lung injury. Am J Respir Crit Care Med 2006;174:187–97.
12. Kallet RH. A Comprehensive Review of Prone Position in ARDS. Respir Care 2015;60:1660–87.
13. Scholten EL, Beitler JR, Prisk GK, Malhotra A. Treatment of ARDS With Prone Positioning. Chest 2017;151:215–24.
14. Prone Positioning in Severe Acute Respiratory Distress Syndrome | NEJM [Internet]. [cited 2020 Mar 24];Available from: https://www.nejm.org/doi/full/10.1056/nejmoa1214103

15. Pickham D, Berte N, Pihulic M, Valdez A, Mayer B, Desai M. Effect of a wearable patient sensor on care delivery for preventing pressure injuries in acutely ill adults: A pragmatic randomized clinical trial (LS-HAPI study). International Journal of Nursing Studies 2018;80:12–9.
16. Schutt SC, Tarver C, Pezzani M. Pilot study: Assessing the effect of continual position monitoring technology on compliance with patient turning protocols. Nurs Open 2017;5:21–8.
