# Supplementary material for: Assessment of Awake Prone Positioning in Hospitalized Adults With COVID-19: A Nonrandomized Controlled Trial
Source: JAMA Intern Med. 2022 Apr 18;182(6):612–21. doi: 10.1001/jamainternmed.2022.1070 (PMC9016608; doi:10.1001/jamainternmed.2022.1070)
Supplement: Supplement 2. — eMethods 1. Enrollment, Allocation, and Data Extraction eMethods 2. Materials Provided to the Clinical Staff Assisting With the Study eMethods 3. Regulatory Considerations eResults 1. Worst Status on Day 5, Adjusted eResults 2. Worst Status on Day 0 eResults 3. Worst Status on Day 1 eResults 4. Worst Status on Day 2 eResults 5. Worst Status on Day 3 eResults 6. Worst Status on Day 4 eTable 1. WHO Ordinal Scale at Enrollment (Day 0) eTable 2. WHO Ordinal Scale at Day 1 eTable 3. WHO Ordinal Scale at Day 2 eTable 4. WHO Ordinal Scale at Day 3 eTable 5. WHO Ordinal Scale at Day 4 eTable 6. Worst Outcome at Day 5 by Assigned Treatment (Table 3 in Statistical Report) eTable 7. Worst Outcome on WHO Ordinal Scale at Day 14 by Assigned Treatment (Table 6 in Statistical Report) eTable 8. Worst Outcome on Main Ordinal Scale at Day 14 by Assigned Treatment (Table 9 in Statistical Report) eTable 9. Worst Outcome on WHO Ordinal Scale at Day 28 by Assigned Treatment (Table 8 in Statistical Report) eTable 10. Worst Outcome on Main Ordinal Scale at Day 28 by Assigned Treatment (Table 10 in Statistical Report) eFigure 1. Average Daily Prone Time for All Participants With Nursing Data Available by Treatment Assignment (Figure 4 in Statistical Report) eFigure 2. Enrollment Time by Treatment Interaction eFigure 3. Baseline Severity by Treatment Interaction eFigure 4. Age by Treatment Interaction eFigure 5. Sex by Treatment Interaction eFigure 6. Race by Treatment Interaction eFigure 7. Elixhauser Score by Treatment Interaction eFigure 8. Site by Treatment Interaction [file jamainternmed-e221070-s002.pdf]

# Supplementary Online Content

Qian ET, Gatto CL, Amusina O, et al; Vanderbilt Learning Healthcare System Platform Investigators. Assessment of awake prone positioning in hospitalized adults with COVID-19: a nonrandomized controlled trial. *JAMA Intern Med*. Published online April 18, 2022. doi:10.1001/jamainternmed.2022.1070

**eMethods 1.** Enrollment, Allocation, and Data Extraction

**eMethods 2.** Materials Provided to the Clinical Staff Assisting With the Study

**eMethods 3.** Regulatory Considerations

**eResults 1.** Worst Status on Day 5, Adjusted

**eResults 2.** Worst Status on Day 0

**eResults 3.** Worst Status on Day 1

**eResults 4.** Worst Status on Day 2

**eResults 5.** Worst Status on Day 3

**eResults 6.** Worst Status on Day 4

**eTable 1.** WHO Ordinal Scale at Enrollment (Day 0)

**eTable 2.** WHO Ordinal Scale at Day 1

**eTable 3.** WHO Ordinal Scale at Day 2

**eTable 4.** WHO Ordinal Scale at Day 3

**eTable 5.** WHO Ordinal Scale at Day 4

**eTable 6.** Worst Outcome at Day 5 by Assigned Treatment (Table 3 in Statistical Report)

**eTable 7.** Worst Outcome on WHO Ordinal Scale at Day 14 by Assigned Treatment (Table 6 in Statistical Report)

**eTable 8.** Worst Outcome on Main Ordinal Scale at Day 14 by Assigned Treatment (Table 9 in Statistical Report)

**eTable 9.** Worst Outcome on WHO Ordinal Scale at Day 28 by Assigned Treatment (Table 8 in Statistical Report)

**eTable 10.** Worst Outcome on Main Ordinal Scale at Day 28 by Assigned Treatment (Table 10 in Statistical Report)

**eFigure 1.** Average Daily Prone Time for All Participants With Nursing Data Available by Treatment Assignment (Figure 4 in Statistical Report)

**eFigure 2.** Enrollment Time by Treatment Interaction

**eFigure 3.** Baseline Severity by Treatment Interaction

**eFigure 4.** Age by Treatment Interaction

**eFigure 5.** Sex by Treatment Interaction

**eFigure 6.** Race by Treatment Interaction

**eFigure 7.** Elixhauser Score by Treatment Interaction

**eFigure 8.** Site by Treatment Interaction

This supplementary material has been provided by the authors to give readers additional information about their work.

© 2022 Qian ET et al. *JAMA Internal Medicine*.

© 2022 Qian ET et al. *JAMA Internal Medicine*.

**eMethods 1.** Enrollment, Randomization, and Data Extraction

Enrollment and Randomization

*Specific procedures employed at VUMC:* The patient census at 8 AM each morning was reviewed for new, non-invasive supplemental oxygen provision. Any patients on oxygen at 8 AM were allocated according to study procedures and enrolled in the trial. This assignment was communicated to the clinical team by 10 AM for review during the daily “COVID Huddle”. At VUMC, we enrolled 100% of eligible participants (461).

*Specific procedures employed at NorthShore:* COVID census was reviewed twice a day (between 6 AM – 9 AM) and again (between 4 PM – 6 PM) Monday-Friday and mornings Saturday/Sunday by one of the members of the study team. Patients were contacted to obtain a verbal consent after assurance that the treatment team (typically a hospitalist medicine attending or an ICU attending) agreed to patient participation. It was indicated in charts when patients enrolled and trial arm assignment. At NorthShore, 100% of eligible patients were approached, with an enrollment success rate of 71% (56 patients were approached with 40 enrolling and 16 patients declining to have data collected).

*Time from eligibility to enrollment:* Across both sites, average overall patient time from eligibility to enrollment was 11.68 hours. (Statistical Report Table 1)

Data Extraction

VUMC data were obtained via Structured Query Language extraction of an existing operational and analytical data warehouse entirely derived from the electronic health record. Investigators at NorthShore manually collected data to include measures that sufficiently inform all primary and secondary study outcomes.

Race and ethnicity were self-reported by patients or their surrogates upon patient registration. This data was extracted with other variables as indicated above. Given that outcomes from hospitalized COVID-19 patients may differ based on race and ethnicity, reporting these demographics was important.

© 2022 Qian ET et al. *JAMA Internal Medicine*.

**eMethods 2.** Materials Provided to the Clinical Staff Assisting With the Study

Proning Trial Summary

## VUMC Patient Proning Study in COVID-19+ Patients
## GUIDELINES FOR PROVIDERS

**Brief Summary:** During the COVID pandemic, there have been a number of anecdotal reports and small observational studies suggesting that prone-positioning has demonstrated an improvement in oxygenation. This Study will assess if prone-positioning in hypoxic, non-ventilated COVID (+) patients will have sustained improvement in clinical outcomes.

**Eligibility:** All patients admitted to VUMC with a positive COVID-19 test result who require supplemental oxygen but are not mechanically ventilated

**Assignments:**

- EVEN MRNs: usual care
- ODD MRNs: proning recommendation and reminders

**Proning Considerations:**

- **Be mindful of any injuries/conditions** that could compromise a patient's ability to prone, such as:
    - Pregnancy
    - Open abdomen
    - Recent surgery (specifically within 14-15 days of tracheal surgery, sternotomy, facial trauma)
    - Unstable fracture of spine, femur, or pelvis
- Always strive to **protect the integrity of line placements and similar patient monitoring/assisting devices.**
- For patients able and willing to prone, encourage them to **prone as often and consistently as they are able, ideally at least 3 hours at a time 4 times a day.**
- Some patients might struggle with boredom and/or fatigue while proning. Feel free to **recommend alternative entertainment** like listening to music, podcasts, or audiobooks.
- To help **maximize patient comfort while proning,** consider putting a pillow under pelvis/abdomen and under the lower legs, as this may alleviate lower back strain.

**If you have questions regarding the study, please reach out to one of the following study PIs:**

Todd Rice: todd.rice@vumc.org
Eddie Qian: edward.t.qian@vumc.org

**Thank you** for your continued efforts and commitment!

VUMC IRB# 200727 Clinical Trials Record# NCT04359797

© 2022 Qian ET et al. *JAMA Internal Medicine*.

Be On Your Belly Signage

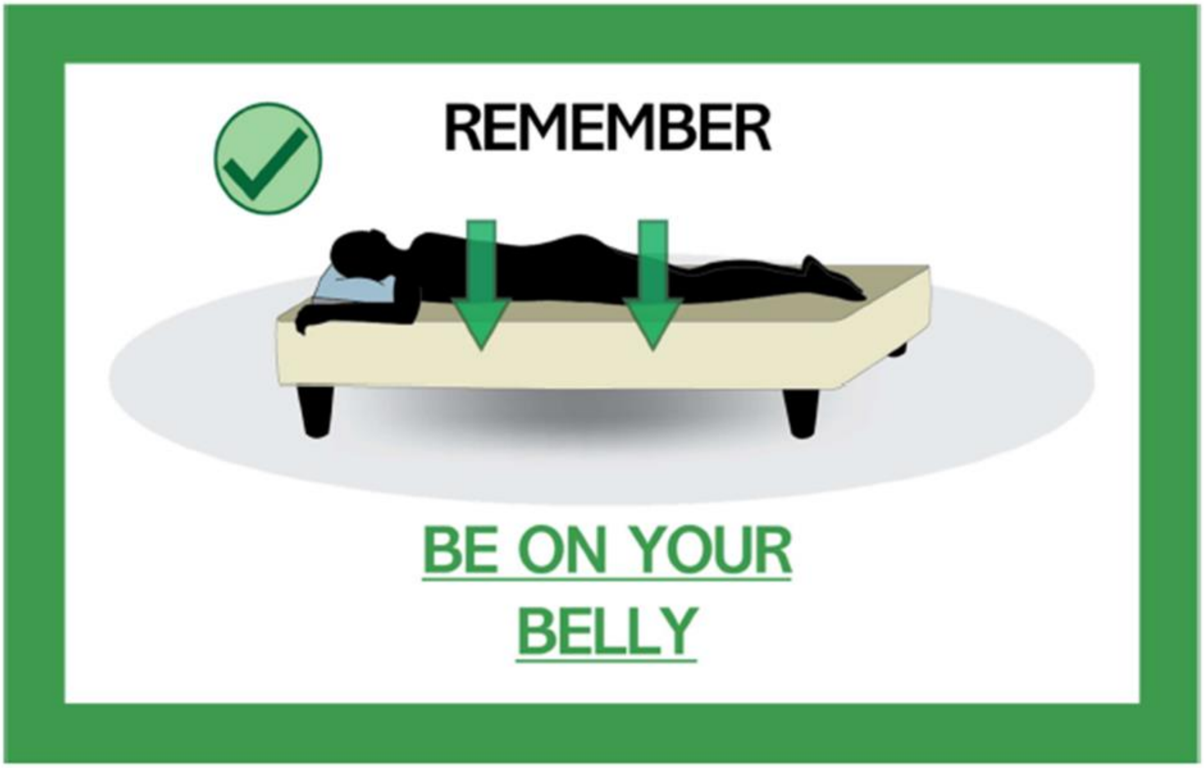


© 2022 Qian ET et al. *JAMA Internal Medicine*.

**eMethods 3**. Regulatory

Regulatory Considerations--VUMC and NorthShore

Given the fact that the practice of awake prone positioning was being employed on a national scale very early on in the pandemic, combined with the fact that the usual care arm allowed awake prone positioning at the discretion of the primary medical team, this trial was presented to the IRB as no greater than minimal risk as patients were able to experience awake prone positioning as a matter of routine course during inpatient treatment for COVID-19.
At Vanderbilt University Medical Center, waiver of informed consent was sought due to the impracticality of consenting patients without imparting undue bias by informing them about awake prone positioning. Awareness of research engagement would necessarily shift the focus of the intervention away from real world effectiveness as delivered in routine care. For example, making patients aware of research examining prone positioning might encourage patients receiving usual care to assume a prone position when they would otherwise not have done so even if their primary team had not recommended it. As indicated, NorthShore University HealthSystem employed a statement confirming the patient's knowledge that their data would be collected for research purposes delivered to each patient on enrollment with the ascertainment of the patient's verbal consent. The statement used did not mention the awake prone positioning intervention and was as follows:

> "We would like to collect information related to your COVID-related care and your medical records for a research study that will help us learn how to improve on care for other patients during this Pandemic"

© 2022 Qian ET et al. *JAMA Internal Medicine*.

**eResults 1.** Worst Status on Day 5, Adjusted

- Re-did the primary analysis with only Vanderbilt subjects as a sensitivity analysis
- Fit a Bayesian proportional odds model, comparing worst outcome on day 5 between the assigned treatment arms (prone and Usual Care)
- Included covariates: Age, sex, race-ethnicity, BMI, Elixhauser score, baseline severity, enrollment time (May-Sept or Oct-Dec)
- Multiple imputation was used to impute missing data for BMI, baseline severity, and Elixhauser score

```
## stackMI(formula = total_outcome ~ Treatment + Baseline + Month +
##     Age + Sex + Race + BMI + Elix, fitter = blrm, xtrans = mi,
##     data = vanderbilt, iter = 4000, warmup = 2000, chain = 4,
##     loo = T, cores = 4)
```

| Median | Lower | Upper |
|---|---|---|
| 1.52 | 1.08 | 2.17 |

| Posterior Probabilty (Prone) |
|---|
| 0.009875 |

The odds of having a worse outcome on day 5 at Vanderbilt are 1.52 (1.08, 2.17) times greater for patients in the prone group compared to the Usual Care group (p = 0.010)

© 2022 Qian ET et al. *JAMA Internal Medicine*.

**eResults 2.** Worst Status on Day 0

## Worst status on day 0, unadjusted

Fit a Bayesian proportional odds model, comparing worst outcome on day 0 between the assigned treatment arms (prone and Usual Care)

- No covariates added to the model

```
## blrm(formula = outcome_day0_c ~ Treatment, data = sec_out_fullb,
##     iter = 4000, chains = 4, warmup = 2000, cores = 4)
```

| Median | Lower | Upper |
|---|---|---|
| 1.01 | 0.68 | 1.5 |

| Posterior Probabilty (Prone) |
|---|
| 0.48 |

The odds of being in a worse outcome rank on day 0 are 1.01 (0.68, 1.50) times greater for patients in the prone group compared to the Usual Care group (p = 0.480)

## Worst status on day 0, adjusted

Fit a Bayesian proportional odds model, comparing worst outcome on day 0 between the assigned treatment arms (prone and Usual Care)

- Included covariates: Age, sex, site, race-ethnicity, BMI, Elixhauser score, baseline severity, enrollment time (May-Sept or Oct-Dec)
- Multiple imputation was used to impute missing data for BMI, baseline severity, and Elixhauser score

```
## stackMI(formula = outcome_day0_c ~ Treatment + baseline_severity +
##     EnrollmentMonth_Group + age + Sex + site + race_ethnicity +
##     bmi + TotalScore, fitter = blrm, xtrans = mi, data = sec_full,
##     iter = 4000, warmup = 2000, chain = 4, loo = T, cores = 4)
```

| Median | Lower | Upper |
|---|---|---|
| 1.02 | 0.63 | 1.63 |

| Posterior Probabilty (Prone) |
|---|
| 0.4733 |

The odds of being in a worse outcome rank on day 0 are 1.02 (0.63, 1.63) times greater for patients in the prone group compared to the Usual Care group (p = 0.473)

© 2022 Qian ET et al. *JAMA Internal Medicine*.

**eResults 3.** Worst Status on Day 1

## Worst status on day 1, unadjusted

Fit a Bayesian proportional odds model, comparing worst outcome on day 1 between the assigned treatment arms (prone and Usual Care)

- No covariates added to the model

```
## blrm(formula = outcome_day1_c ~ Treatment, data = sec_out_fullb,
##     iter = 4000, chains = 4, warmup = 2000, cores = 4)
```

| Median | Lower | Upper |
|---|---|---|
| 1.16 | 0.83 | 1.62 |

| Posterior Probabilty (Prone) |
|---|
| 0.18775 |

The odds of being in a worse outcome rank on day 1 are 1.16 (0.83, 1.62) times greater for patients in the prone group compared to the Usual Care group (p = 0.188)

## Worst status on day 1, adjusted

Fit a Bayesian proportional odds model, comparing worst outcome on day 1 between the assigned treatment arms (prone and Usual Care)

- Included covariates: Age, sex, site, race-ethnicity, BMI, Elixhauser score, baseline severity, enrollment time (May-Sept or Oct-Dec)
- Multiple imputation was used to impute missing data for BMI, baseline severity, and Elixhauser score

| Median | Lower | Upper |
|---|---|---|
| 1.16 | 0.81 | 1.67 |

| Posterior Probabilty (Prone) |
|---|
| 0.2084 |

The odds of being in a worse outcome rank on day 1 are 1.16 (0.81, 1.67) times greater for patients in the prone group compared to the Usual Care group (p = 0.208)

© 2022 Qian ET et al. *JAMA Internal Medicine*.

**eResults 4.** Worst Status on Day 2

## Worst status on day 2, unadjusted

- Fit a Bayesian proportional odds model, comparing worst outcome on day 2 between the assigned treatment arms (prone and Usual Care)
- No covariates added to the model

```
## blrm(formula = outcome_day2_c ~ Treatment, data = sec_out_fullb,
##     iter = 4000, chains = 4, warmup = 2000, cores = 4)
```

| Median | Lower | Upper |
|---|---|---|
| 1.07 | 0.78 | 1.47 |

| Posterior Probabilty (Prone) |
|---|
| 0.33375 |

The odds of being in a worse outcome rank on day 2 are 1.07 (0.78, 1.47) times greater for patients in the prone group compared to the Usual Care group (p = 0.334)

## Worst status on day 2, adjusted

Fit a Bayesian proportional odds model, comparing worst outcome on day 2 between the assigned treatment arms (prone and Usual Care)

- Included covariates: Age, sex, site, race-ethnicity, BMI, Elixhauser score, baseline severity, enrollment time (May-Sept or Oct-Dec)
- Multiple imputation was used to impute missing data for BMI, baseline severity, and Elixhauser score

```
## stackMI(formula = outcome_day2_c ~ Treatment + baseline_severity +
##     EnrollmentMonth_Group + age + Sex + site + race_ethnicity +
##     bmi + TotalScore, fitter = blrm, xtrans = mi, data = sec_full,
##     iter = 4000, warmup = 2000, chain = 4, loo = T, cores = 4)
```

| Median | Lower | Upper |
|---|---|---|
| 1.06 | 0.74 | 1.46 |

| Posterior Probabilty (Prone) |
|---|
| 0.3752 |

The odds of being in a worse outcome rank on day 2 are 1.06 (0.74, 1.46) times greater for patients in the prone group compared to the Usual Care group (p = 0.375)

© 2022 Qian ET et al. *JAMA Internal Medicine*.

**eResults 5.** Worst Status on Day 3

## Worst status on day 3, unadjusted

Fit a Bayesian proportional odds model, comparing worst outcome on day 3 between the assigned treatment arms (prone and Usual Care)

- No covariates added to the model

```
## blrm(formula = outcome_day3_c ~ Treatment, data = sec_out_fullb,
##      iter = 4000, chains = 4, warmup = 2000, cores = 4)
```

| Median | Lower | Upper |
|---|---|---|
| 1.17 | 0.85 | 1.59 |

| Posterior Probabilty (Prone) |
|---|
| 0.16425 |

The odds of being in a worse outcome rank on day 3 are 1.17 (0.85, 1.59) times greater for patients in the prone group compared to the Usual Care group (p = 0.164)

## Worst status on day 3, adjusted

Fit a Bayesian proportional odds model, comparing worst outcome on day 3 between the assigned treatment arms (prone and Usual Care)

- Included covariates: Age, sex, site, race-ethnicity, BMI, Elixhauser score, baseline severity, enrollment time (May-Sept or Oct-Dec)
- Multiple imputation was used to impute missing data for BMI, baseline severity, and Elixhauser score

```
## stackMI(formula = outcome_day3_c ~ Treatment + baseline_severity +
##     EnrollmentMonth_Group + age + Sex + site + race_ethnicity +
##     bmi + TotalScore, fitter = blrm, xtrans = mi, data = sec_full,
##     iter = 4000, warmup = 2000, chain = 4, loo = T, cores = 4)
```

| Median | Lower | Upper |
|---|---|---|
| 1.22 | 0.88 | 1.7 |

| Posterior Probabilty (Prone) |
|---|
| 0.120675 |

The odds of being in a worse outcome rank on day 3 are 1.22 (0.88, 1.70) times greater for patients in the prone group compared to the Usual Care group (p = 0.121)

© 2022 Qian ET et al. *JAMA Internal Medicine*.

**eResults 6.** Worst Status on Day 4

## Worst status on day 4, unadjusted

Fit a Bayesian proportional odds model, comparing worst outcome on day 4 between the assigned treatment arms (prone and Usual Care)

- No covariates added to the model

```
## blrm(formula = outcome_day4_c ~ Treatment, data = sec_out_fullb,
##     iter = 4000, chains = 4, warmup = 2000, cores = 4)
```

| Median | Lower | Upper |
|---|---|---|
| 1.28 | 0.94 | 1.77 |

| Posterior Probabilty (Prone) |
|---|
| 0.0615 |

The odds of being in a worse outcome rank on day 4 are 1.28 (0.94, 1.77) times greater for patients in the prone group compared to the Usual Care group (p = 0.062)

## Worst status on day 4, adjusted

Fit a Bayesian proportional odds model, comparing worst outcome on day 4 between the assigned treatment arms (prone and Usual Care)

- Included covariates: Age, sex, site, race-ethnicity, BMI, Elixhauser score, baseline severity, enrollment time (May-Sept or Oct-Dec)
- Multiple imputation was used to impute missing data for BMI, baseline severity, and Elixhauser score

```
## stackMI(formula = outcome_day4_c ~ Treatment + baseline_severity +
##     EnrollmentMonth_Group + age + Sex + site + race_ethnicity +
##     bmi + TotalScore, fitter = blrm, xtrans = mi, data = sec_full,
##     iter = 4000, warmup = 2000, chain = 4, loo = T, cores = 4)
```

| Median | Lower | Upper |
|---|---|---|
| 1.39 | 0.99 | 1.94 |

| Posterior Probabilty (Prone) |
|---|
| 0.027725 |

The odds of being in a worse outcome rank on day 4 are 1.39 (0.99, 1.94) times greater for patients in the prone group compared to the Usual Care group (p = 0.028)

© 2022 Qian ET et al. *JAMA Internal Medicine*.

**eTable 1.** WHO Ordinal Scale at Enrollment (Day 0)

**Day 0 (Enrollment Day)**

| | Overall | Usual Care | Prone |
|---|---|---|---|
| n | 501 | 243 | 258 |
| Day 0 (%) | | | |
| Discharged | 0 ( 0.0) | 0 ( 0.0) | 0 ( 0.0) |
| Room Air | 0 ( 0.0) | 0 ( 0.0) | 0 ( 0.0) |
| Standard Nasal Cannula | 369 (73.7) | 181 (74.5) | 188 (72.9) |
| High Flow Nasal Cannula | 52 (10.4) | 17 ( 7.0) | 35 (13.6) |
| Non-Invasive Ventilation | 73 (14.6) | 42 (17.3) | 31 (12.0) |
| Mechanical Ventilation | 3 ( 0.6) | 2 ( 0.8) | 1 ( 0.4) |
| ECMO | 0 ( 0.0) | 0 ( 0.0) | 0 ( 0.0) |
| Death | 4 ( 0.8) | 1 ( 0.4) | 3 ( 1.2) |

© 2022 Qian ET et al. *JAMA Internal Medicine*.

**eTable 2.** WHO Ordinal Scale at Day 1

**Day 1**

| | Overall | Usual Care | Prone |
|---|---|---|---|
| n | 501 | 243 | 258 |
| Day 1 (%) | | | |
| Discharged | 24 ( 4.8) | 8 ( 3.3) | 16 ( 6.2) |
| Room Air | 44 ( 8.8) | 25 (10.3) | 19 ( 7.4) |
| Standard Nasal Cannula | 299 (59.7) | 153 (63.0) | 146 (56.6) |
| High Flow Nasal Cannula | 43 ( 8.6) | 11 ( 4.5) | 32 (12.4) |
| Non-Invasive Ventilation | 70 (14.0) | 38 (15.6) | 32 (12.4) |
| Mechanical Ventilation | 13 ( 2.6) | 5 ( 2.1) | 8 ( 3.1) |
| ECMO | 1 ( 0.2) | 1 ( 0.4) | 0 ( 0.0) |
| Death | 7 ( 1.4) | 2 ( 0.8) | 5 ( 1.9) |

© 2022 Qian ET et al. *JAMA Internal Medicine*.

**eTable 3.** WHO Ordinal Scale at Day 2

**Day 2**

| | Overall | Usual Care | Prone |
|---|---|---|---|
| n | 501 | 243 | 258 |
| Day 2 (%) | | | |
| Discharged | 83 (16.6) | 39 (16.0) | 44 (17.1) |
| Room Air | 35 ( 7.0) | 16 ( 6.6) | 19 ( 7.4) |
| Standard Nasal Cannula | 248 (49.5) | 128 (52.7) | 120 (46.5) |
| High Flow Nasal Cannula | 36 ( 7.2) | 13 ( 5.3) | 23 ( 8.9) |
| Non-Invasive Ventilation | 67 (13.4) | 35 (14.4) | 32 (12.4) |
| Mechanical Ventilation | 17 ( 3.4) | 8 ( 3.3) | 9 ( 3.5) |
| ECMO | 1 ( 0.2) | 1 ( 0.4) | 0 ( 0.0) |
| Death | 14 ( 2.8) | 3 ( 1.2) | 11 ( 4.3) |

© 2022 Qian ET et al. *JAMA Internal Medicine*.

**eTable 4.** WHO Ordinal Scale at Day 3

**Day 3**

| | Overall | Usual Care | Prone |
|---|---|---|---|
| n | 501 | 243 | 258 |
| Day 3 (%) | | | |
| Discharged | 137 (27.3) | 66 (27.2) | 71 (27.5) |
| Room Air | 34 ( 6.8) | 18 ( 7.4) | 16 ( 6.2) |
| Standard Nasal Cannula | 192 (38.3) | 100 (41.2) | 92 (35.7) |
| High Flow Nasal Cannula | 40 ( 8.0) | 16 ( 6.6) | 24 ( 9.3) |
| Non-Invasive Ventilation | 58 (11.6) | 29 (11.9) | 29 (11.2) |
| Mechanical Ventilation | 22 ( 4.4) | 9 ( 3.7) | 13 ( 5.0) |
| ECMO | 1 ( 0.2) | 1 ( 0.4) | 0 ( 0.0) |
| Death | 17 ( 3.4) | 4 ( 1.6) | 13 ( 5.0) |

© 2022 Qian ET et al. *JAMA Internal Medicine*.

**eTable 5.** WHO Ordinal Scale at Day 4

**Day 4**

| | Overall | Usual Care | Prone |
|---|---|---|---|
| n | 501 | 243 | 258 |
| Day 4 (%) | | | |
| Discharged | 173 (34.5) | 89 (36.6) | 84 (32.6) |
| Room Air | 32 ( 6.4) | 16 ( 6.6) | 16 ( 6.2) |
| Standard Nasal Cannula | 154 (30.7) | 77 (31.7) | 77 (29.8) |
| High Flow Nasal Cannula | 39 ( 7.8) | 16 ( 6.6) | 23 ( 8.9) |
| Non-Invasive Ventilation | 54 (10.8) | 27 (11.1) | 27 (10.5) |
| Mechanical Ventilation | 24 ( 4.8) | 10 ( 4.1) | 14 ( 5.4) |
| ECMO | 2 ( 0.4) | 1 ( 0.4) | 1 ( 0.4) |
| Death | 23 ( 4.6) | 7 ( 2.9) | 16 ( 6.2) |

© 2022 Qian ET et al. *JAMA Internal Medicine*.

**eTable 6.** Worst Outcome at Day 5 by Assigned Treatment (Table 3 in Statistical Report)

| | Overall | Usual Care | Prone |
|---|---|---|---|
| n | 501 | 243 | 258 |
| Categorical Outcome (%) | | | |
| Discharged | 208 (41.5) | 109 (44.9) | 99 (38.4) |
| Room Air | 30 ( 6.0) | 15 ( 6.2) | 15 ( 5.8) |
| Standard Nasal Cannula | 122 (24.4) | 57 (23.5) | 65 (25.2) |
| High Flow Nasal Cannula | 32 ( 6.4) | 16 ( 6.6) | 16 ( 6.2) |
| Non-Invasive Ventilation | 50 (10.0) | 26 (10.7) | 24 ( 9.3) |
| Mechanical Ventilation | 29 ( 5.8) | 10 ( 4.1) | 19 ( 7.4) |
| ECMO | 2 ( 0.4) | 1 ( 0.4) | 1 ( 0.4) |
| Death | 28 ( 5.6) | 9 ( 3.7) | 19 ( 7.4) |
| Continuous Outcome (mean (SD)) | 3.00 (2.14) | 2.79 (2.02) | 3.19 (2.24) |
| Continuous Outcome (median [IQR]) | 3.12 [1.00, 4.41] | 2.00 [1.00, 4.29] | 3.13 [1.00, 4.50] |

© 2022 Qian ET et al. *JAMA Internal Medicine*.

**eTable 7.** Worst Outcome on WHO Ordinal Scale at Day 14 by Assigned Treatment (Table 6 in Statistical Report)

| | Overall | Usual Care | Prone |
|---|---|---|---|
| n | 501 | 243 | 258 |
| WHO Ordinal Scale (%) | | | |
| Discharge | 332 (66.3) | 164 (67.5) | 168 (65.1) |
| No supplemental Oxygen | 12 ( 2.4) | 7 ( 2.9) | 5 ( 1.9) |
| Supplemental Oxygen | 26 ( 5.2) | 12 ( 4.9) | 14 ( 5.4) |
| NIV | 17 ( 3.4) | 8 ( 3.3) | 9 ( 3.5) |
| MV or ECMO | 22 ( 4.4) | 13 ( 5.3) | 9 ( 3.5) |
| Death | 90 (18.0) | 38 (15.6) | 52 (20.2) |
| Unknown/Missing | 2 ( 0.4) | 1 ( 0.4) | 1 ( 0.4) |

© 2022 Qian ET et al. *JAMA Internal Medicine*.

**eTable 8.** Worst Outcome on Main Ordinal Scale at Day 14 by Assigned Treatment (Table 9 in Statistical Report)

| | Overall | Usual Care | Prone |
|---|---|---|---|
| n | 501 | 243 | 258 |
| Ordinal Scale (%) | | | |
| Discharge | 332 (66.3) | 164 (67.5) | 168 (65.1) |
| Room Air | 11 ( 2.2) | 7 ( 2.9) | 4 ( 1.6) |
| Low Flow | 23 ( 4.6) | 10 ( 4.1) | 13 ( 5.0) |
| High Flow | 5 ( 1.0) | 3 ( 1.2) | 2 ( 0.8) |
| NIV | 16 ( 3.2) | 7 ( 2.9) | 9 ( 3.5) |
| MV | 17 ( 3.4) | 9 ( 3.7) | 8 ( 3.1) |
| ECMO | 5 ( 1.0) | 4 ( 1.6) | 1 ( 0.4) |
| Death | 90 (18.0) | 38 (15.6) | 52 (20.2) |
| Unknown/Missing | 2 ( 0.4) | 1 ( 0.4) | 1 ( 0.4) |

© 2022 Qian ET et al. *JAMA Internal Medicine*.

**eTable 9.** Worst Outcome on WHO Ordinal Scale at Day 28 by Assigned Treatment (Table 8 in Statistical Report)

## Vanderbilt Participants Only

- Added post hoc

| | Overall | Usual Care | Prone |
|---|---|---|---|
| n | 461 | 222 | 239 |
| WHO Ordinal Scale (%) | | | |
| Discharge | 336 (72.9) | 162 (73.0) | 174 (72.8) |
| No supplemental Oxygen | 2 ( 0.4) | 1 ( 0.5) | 1 ( 0.4) |
| Supplemental Oxygen | 2 ( 0.4) | 1 ( 0.5) | 1 ( 0.4) |
| NIV | 7 ( 1.5) | 4 ( 1.8) | 3 ( 1.3) |
| MV or ECMO | 11 ( 2.4) | 7 ( 3.2) | 4 ( 1.7) |
| Death | 103 (22.3) | 47 (21.2) | 56 (23.4) |

© 2022 Qian ET et al. *JAMA Internal Medicine*.

**eTable 10.** Worst Outcome on Main Ordinal Scale at Day 28 by Assigned Treatment (Table 10 in Statistical Report)

**Vanderbilt Participants Only**

| | Overall | Usual Care | Prone |
|---|---|---|---|
| n | 461 | 222 | 239 |
| Ordinal Scale (%) | | | |
| Discharge | 336 (72.9) | 162 (73.0) | 174 (72.8) |
| Room Air | 2 ( 0.4) | 1 ( 0.5) | 1 ( 0.4) |
| Low Flow | 3 ( 0.7) | 2 ( 0.9) | 1 ( 0.4) |
| High Flow | 2 ( 0.4) | 1 ( 0.5) | 1 ( 0.4) |
| NIV | 4 ( 0.9) | 2 ( 0.9) | 2 ( 0.8) |
| MV | 6 ( 1.3) | 3 ( 1.4) | 3 ( 1.3) |
| ECMO | 5 ( 1.1) | 4 ( 1.8) | 1 ( 0.4) |
| Death | 103 (22.3) | 47 (21.2) | 56 (23.4) |

© 2022 Qian ET et al. *JAMA Internal Medicine*.

**eFigure 1.** Average Daily Prone Time for All Participants With Nursing Data Available by Treatment Assignment (Figure 4 in Statistical Report)

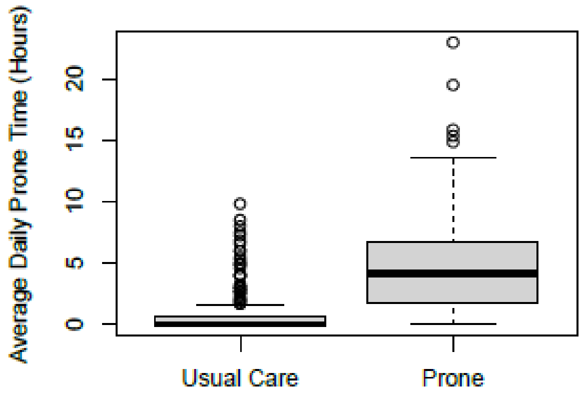


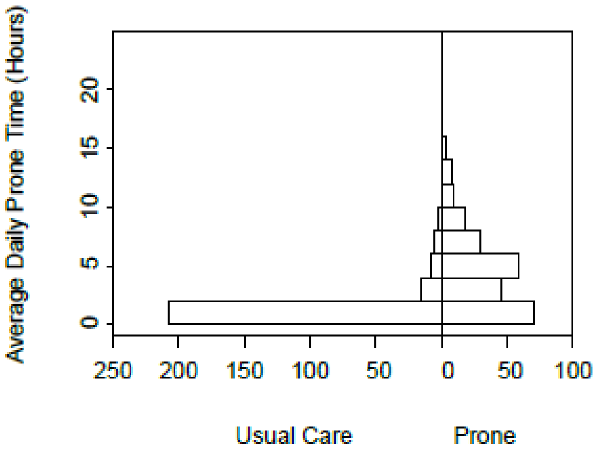


| | Overall | Usual Care | Prone |
|---|---|---|---|
| n | 481 | 238 | 243 |
| Average Daily Prone Time (mean (SD)) | 2.74 (3.57) | 0.80 (1.73) | 4.64 (3.87) |
| Average Daily Prone Time (median [IQR]) | 1.09 [0.00, 4.80] | 0.00 [0.00, 0.66] | 4.18 [1.78, 6.70] |

© 2022 Qian ET et al. *JAMA Internal Medicine*.

**eFigure 2.** Enrollment Time by Treatment Interaction

## Differential Treatment Effects

- Stacking shows what the relative probability of each model being correct is

## Enrollment time by treatment interaction

Comparing model 1 (no interaction) to model 2 (includes treatment by enrollment time interaction)

```
## Method: stacking
## ------
##        weight
## model1 0.607
## model2 0.393
```

The model without the treatment by enrollment time interaction term has a 61% chance of being the correct model, over the model with the interaction term.

## Enrollment Time Period

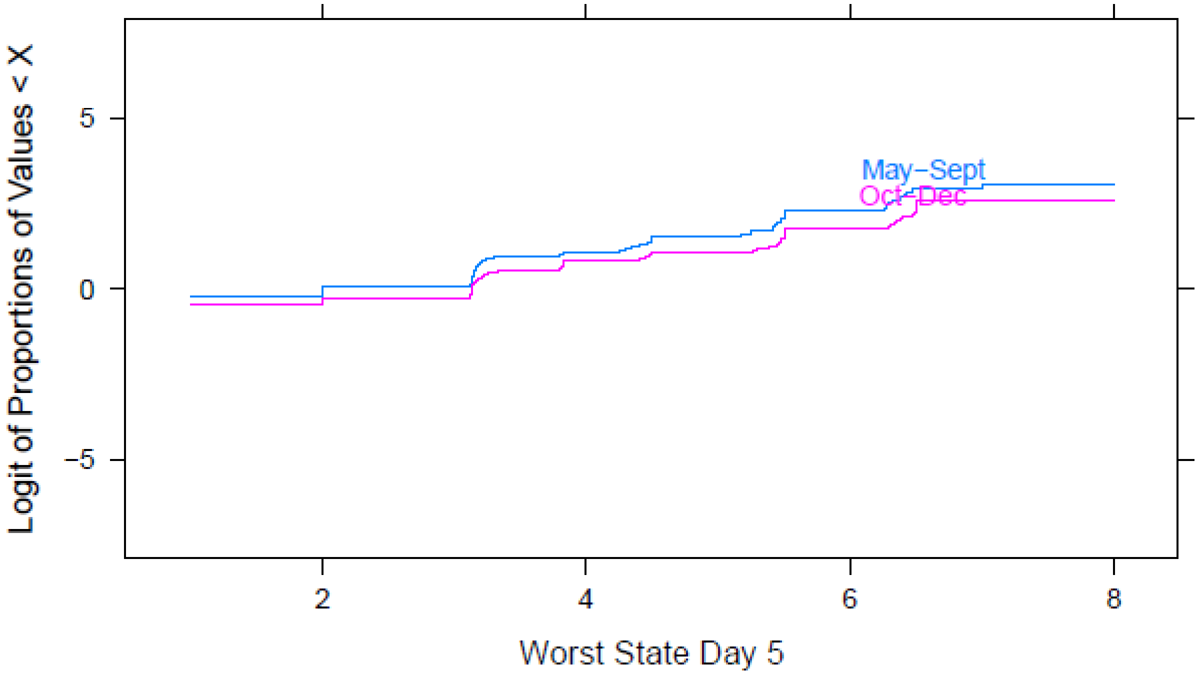


© 2022 Qian ET et al. *JAMA Internal Medicine*.

**eFigure 3.** Baseline Severity by Treatment Interaction

Comparing model 1 (no interaction) to model 2 (includes treatment by baseline severity interaction)

```
## Method: stacking
## ------
##        weight
## model1 0.809
## model2 0.191
```

The model without the treatment by baseline severity interaction term has a 81% chance of being the correct model, over the model with the interaction term.

**Baseline Severity**

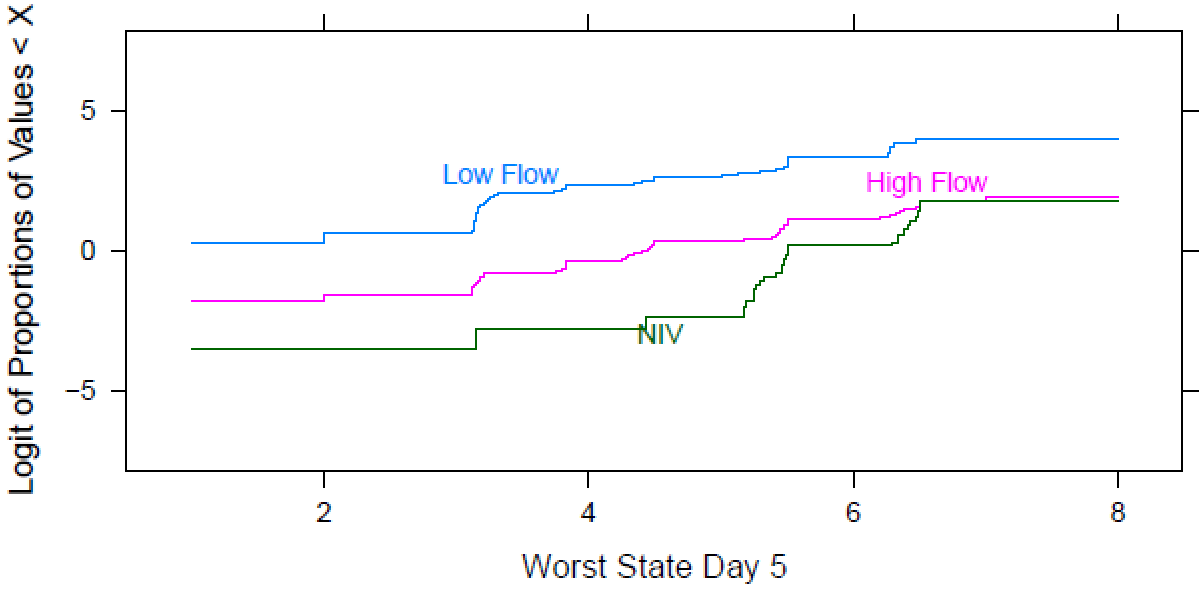


**Baseline Severity: Low Flow**

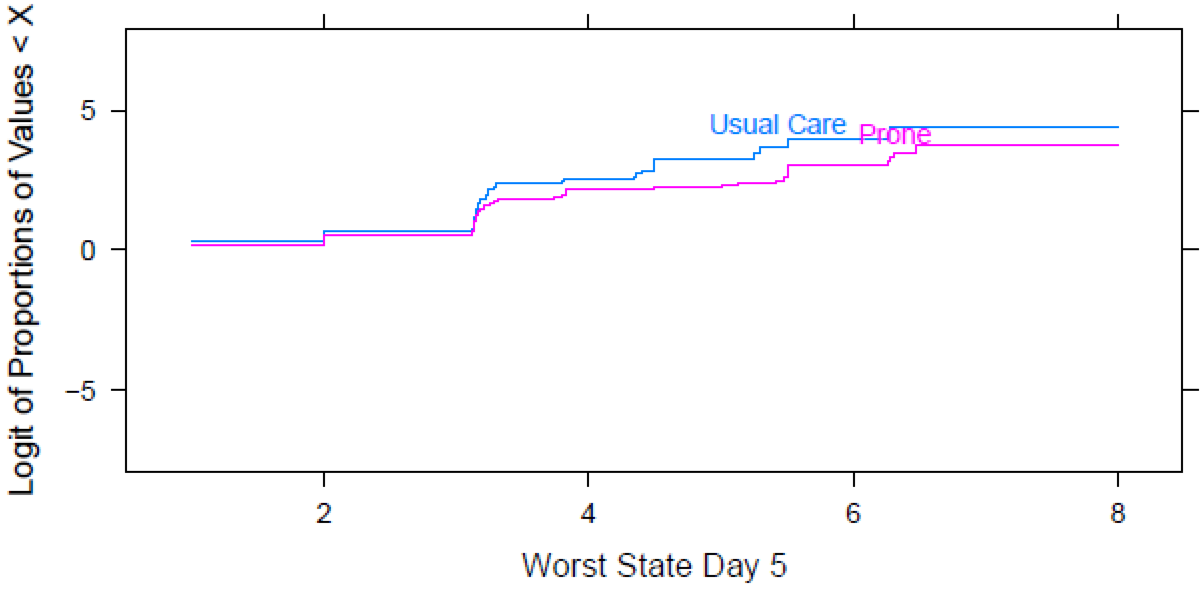


© 2022 Qian ET et al. *JAMA Internal Medicine*.

**Baseline Severity: High Flow**

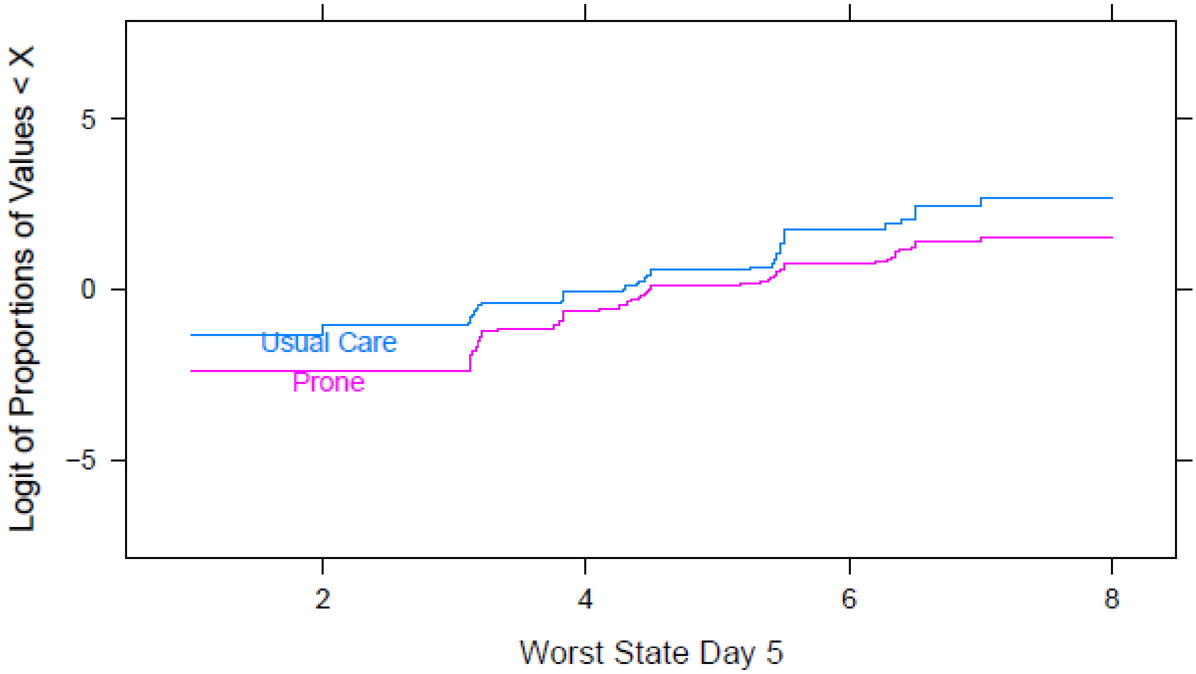


**Baseline Severity: Non-Invasive Ventilation**

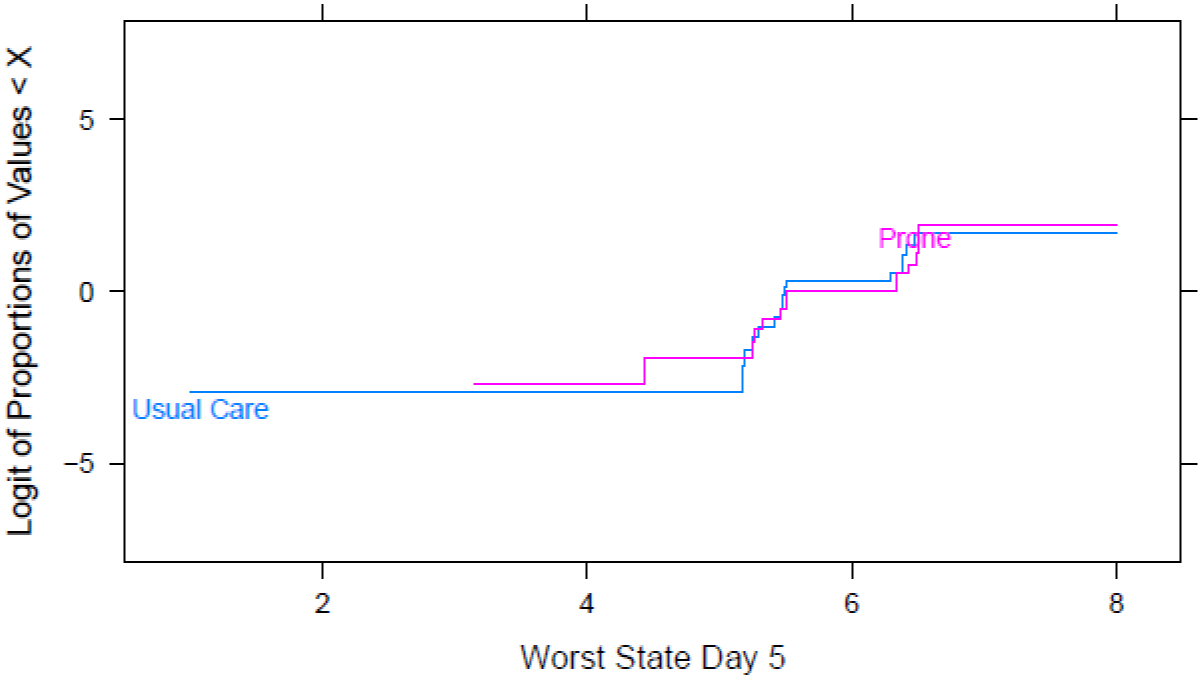


© 2022 Qian ET et al. *JAMA Internal Medicine*.

**eFigure 4.** Age by Treatment Interaction

Comparing model 1 (no interaction) to model 2 (includes treatment by age interaction)

```
## Method: stacking
## ------
##        weight
## model1 0.733
## model2 0.267
```

The model without the treatment by age interaction term has a 73% chance of being the correct model, over the model with the interaction term.

**Age**

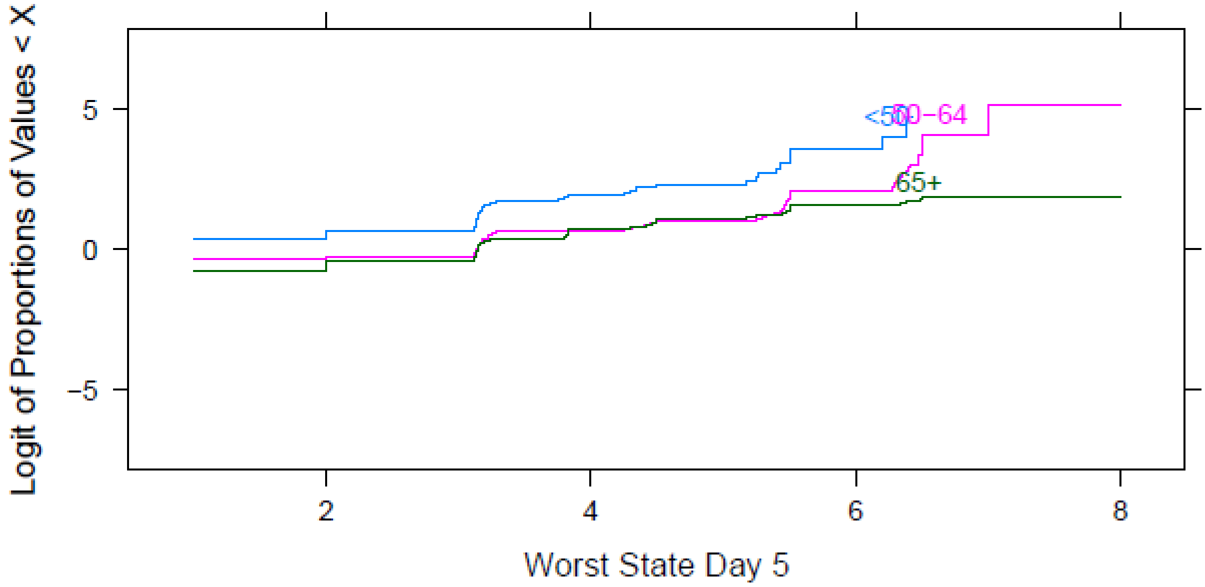


**Age: < 50**

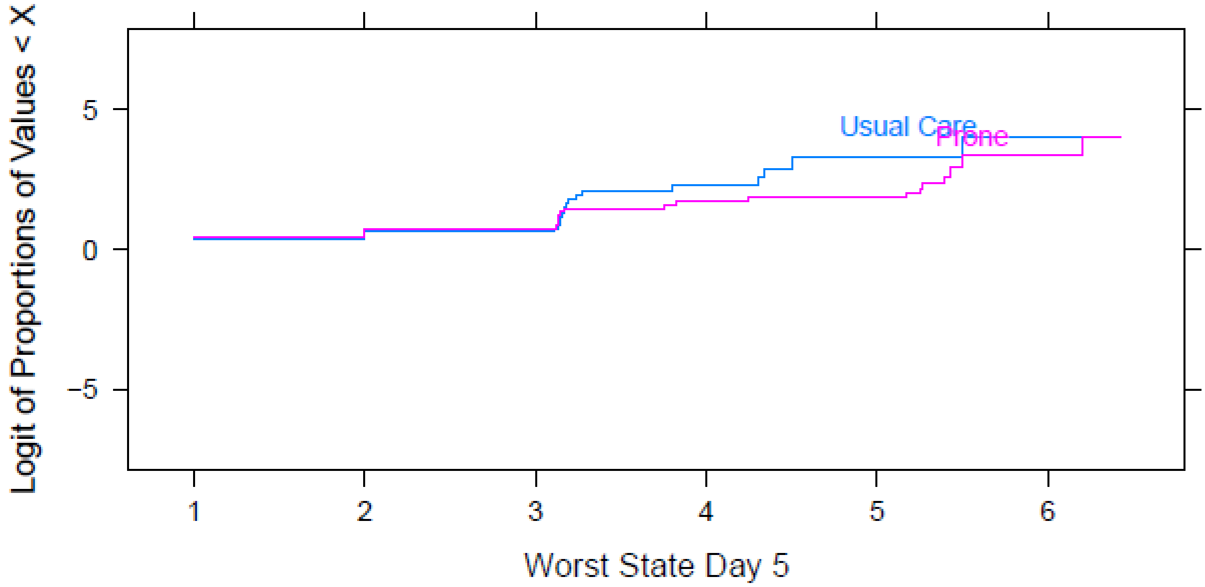


© 2022 Qian ET et al. *JAMA Internal Medicine*.

**Age: 50-64**

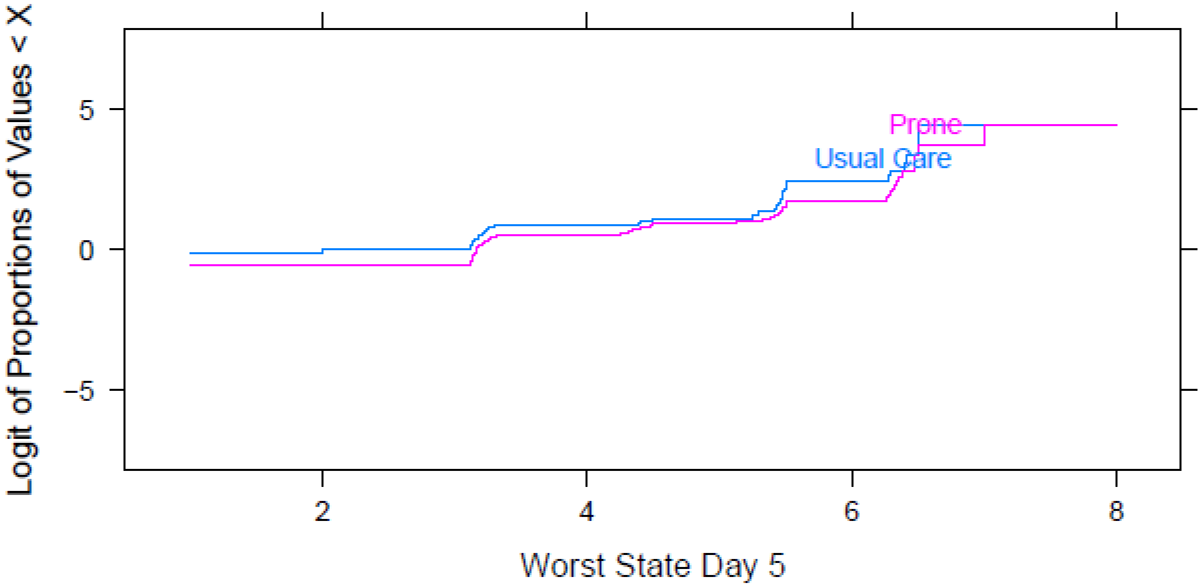


**Age: 65+**

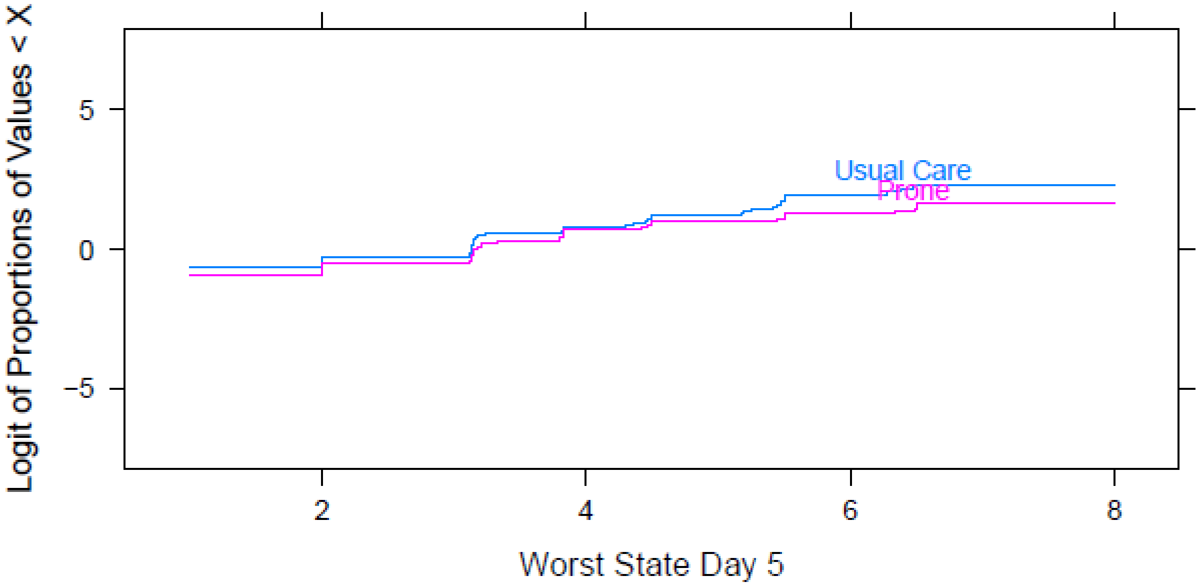


© 2022 Qian ET et al. *JAMA Internal Medicine*.

**eFigure 5.** Sex by Treatment Interaction

Comparing model 1 (no interaction) to model 2 (includes treatment by sex interaction)

```
## Method: stacking
## ------
##         weight
## model1 0.914
## model2 0.086
```

The model without the treatment by sex interaction term has a 91% chance of being the correct model, over the model with the interaction term.

**Sex**

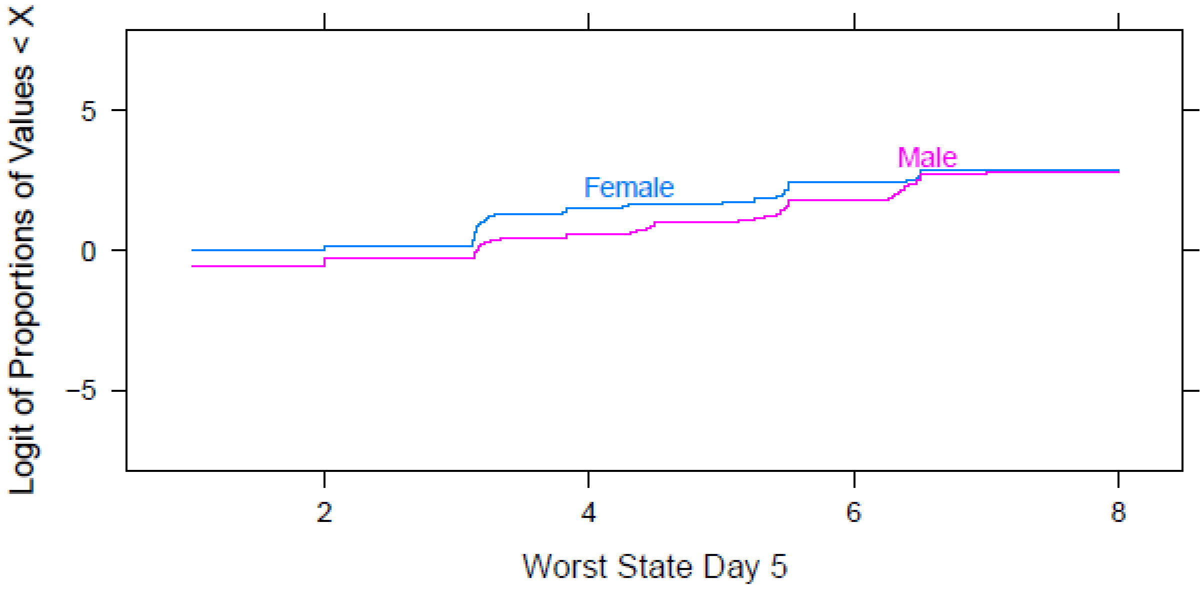


**Sex: Male**

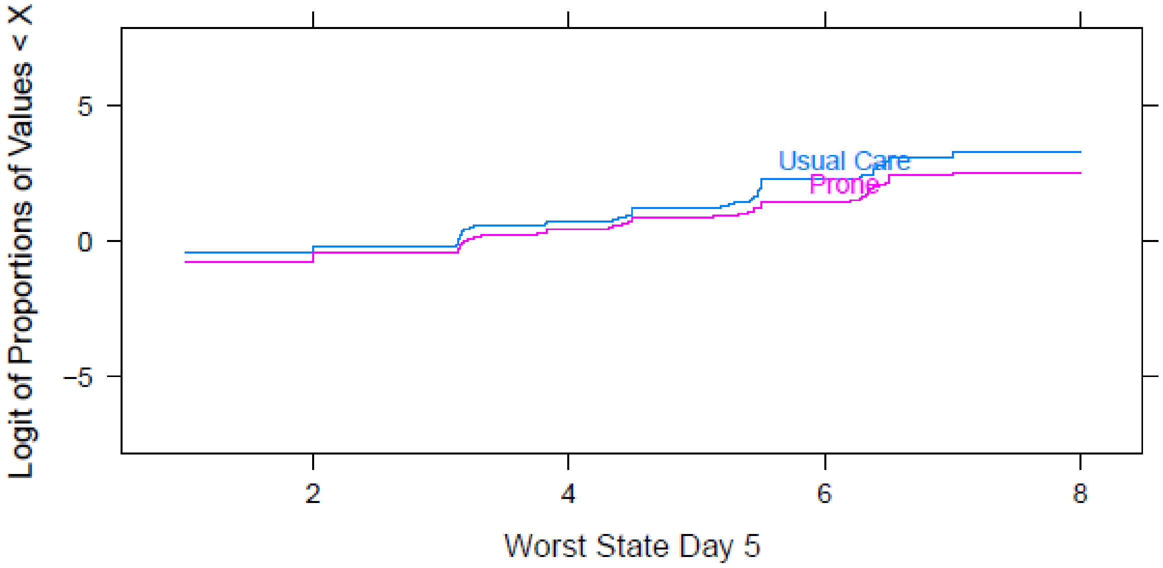


© 2022 Qian ET et al. *JAMA Internal Medicine*.

**Sex: Female**

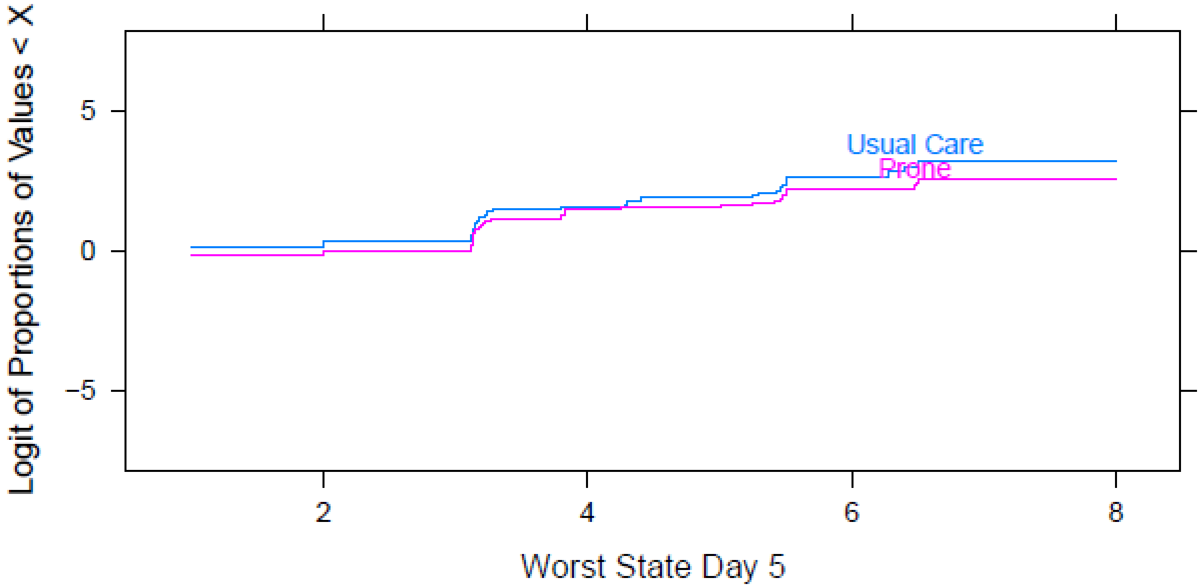


© 2022 Qian ET et al. *JAMA Internal Medicine*.

**eFigure 6.** Race by Treatment Interaction

Comparing model 1 (no interaction) to model 2 (includes treatment by race interaction)

```
## Method: stacking
## ------
##        weight
## model1 0.803
## model2 0.197
```

The model without the treatment by race interaction term has a 80% chance of being the correct model, over the model with the interaction term.

**Race-Ethnicity**

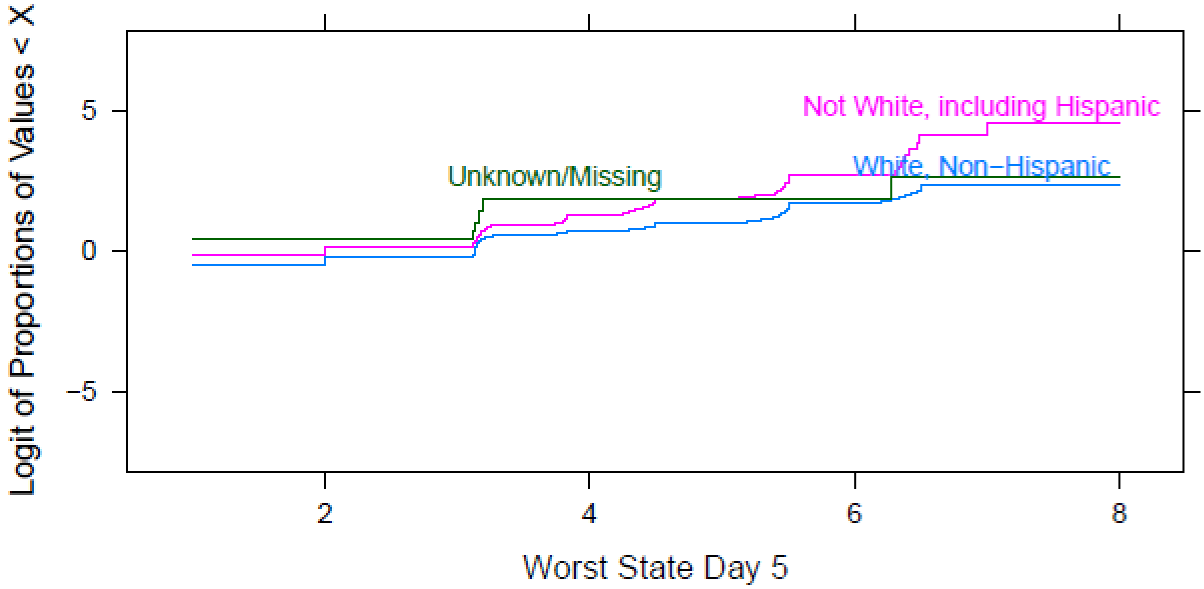


**Race-Ethnicity: White non-Hispanic**

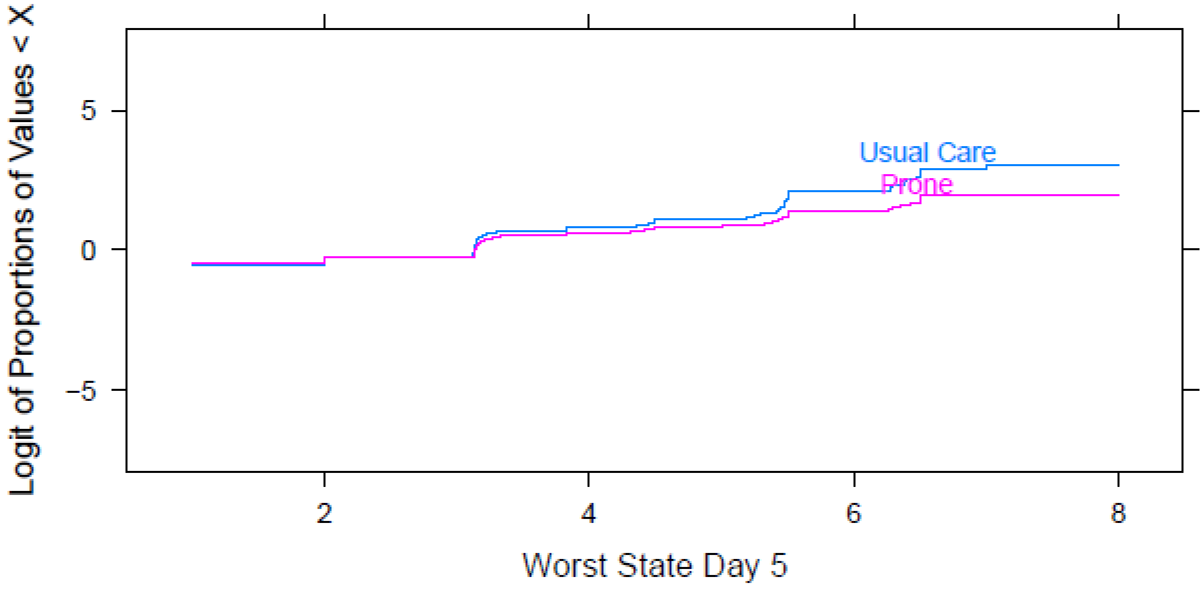


© 2022 Qian ET et al. *JAMA Internal Medicine*.

**Race-Ethnicity: Not White, Including Hispanic**

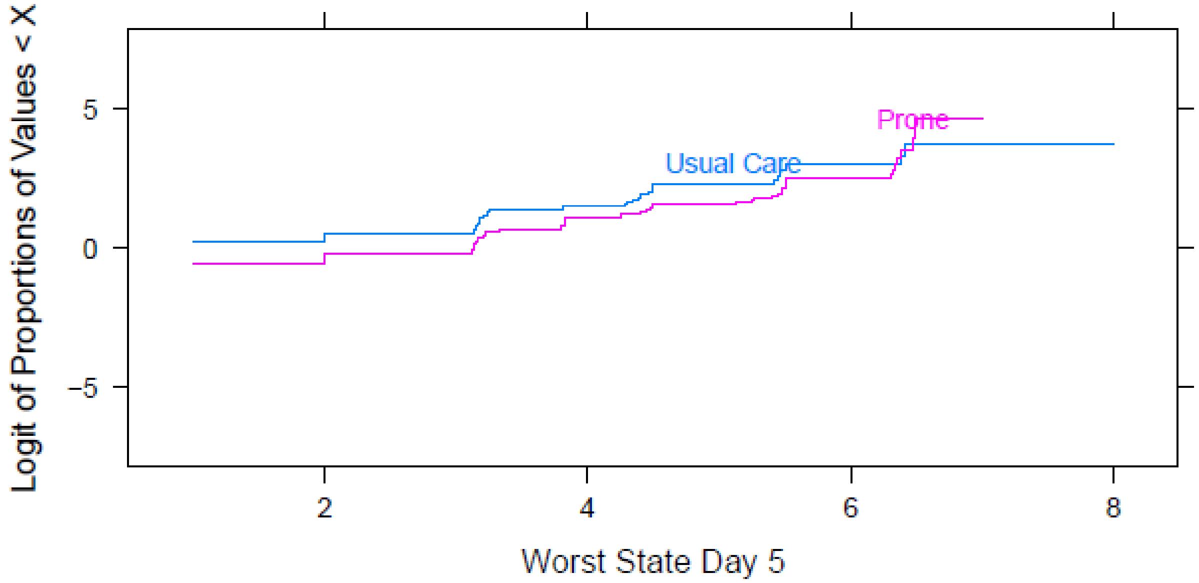


© 2022 Qian ET et al. *JAMA Internal Medicine*.

**eFigure 7.** Elixhauser Score by Treatment Interaction

Comparing model 1 (no interaction) to model 2 (includes treatment by Elixhauser score interaction)

```
## Method: stacking
## ------
##        weight
## model1 0.862
## model2 0.138
```

The model without the treatment by enrollment time interaction term has a 86% chance of being the correct model, over the model with the interaction term.

**Elixhauser Score**

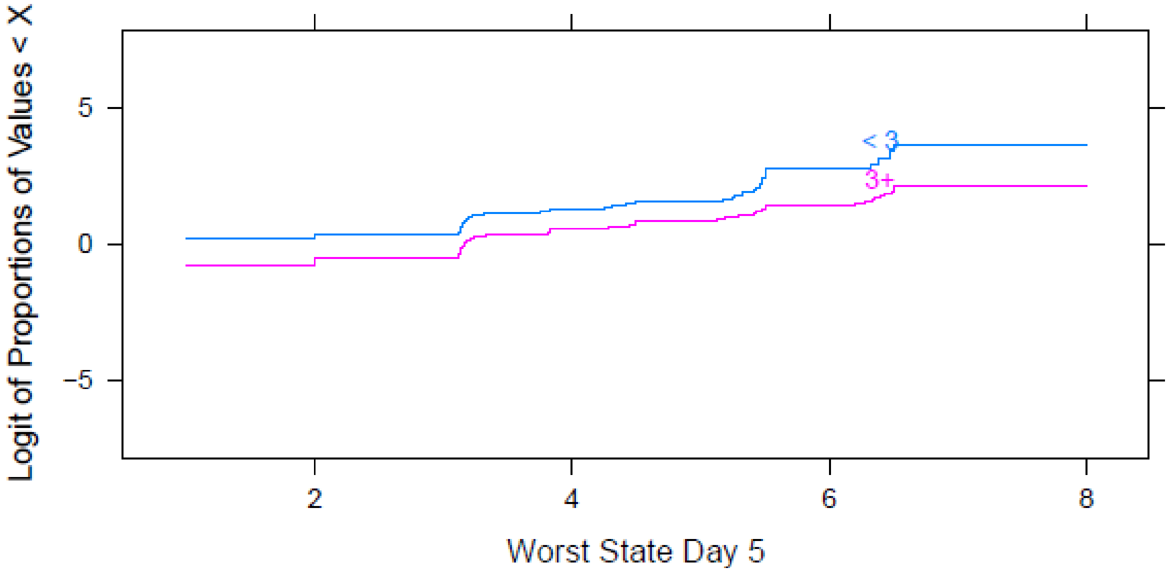


© 2022 Qian ET et al. *JAMA Internal Medicine*.

**Elixhauser Score: < 3**

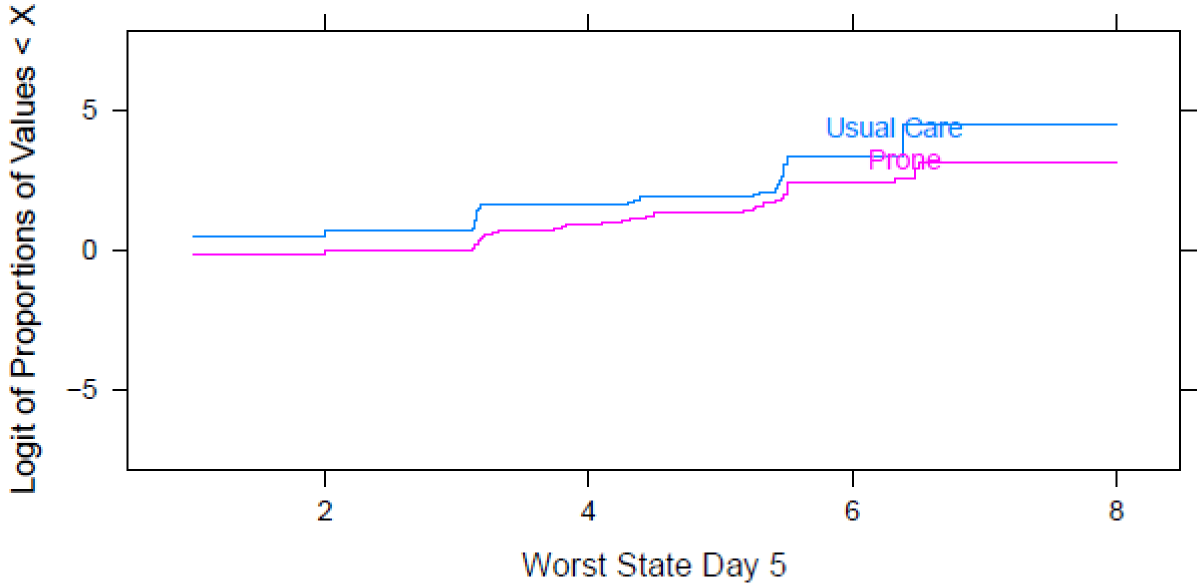


**Elixhauser Score: 3+**

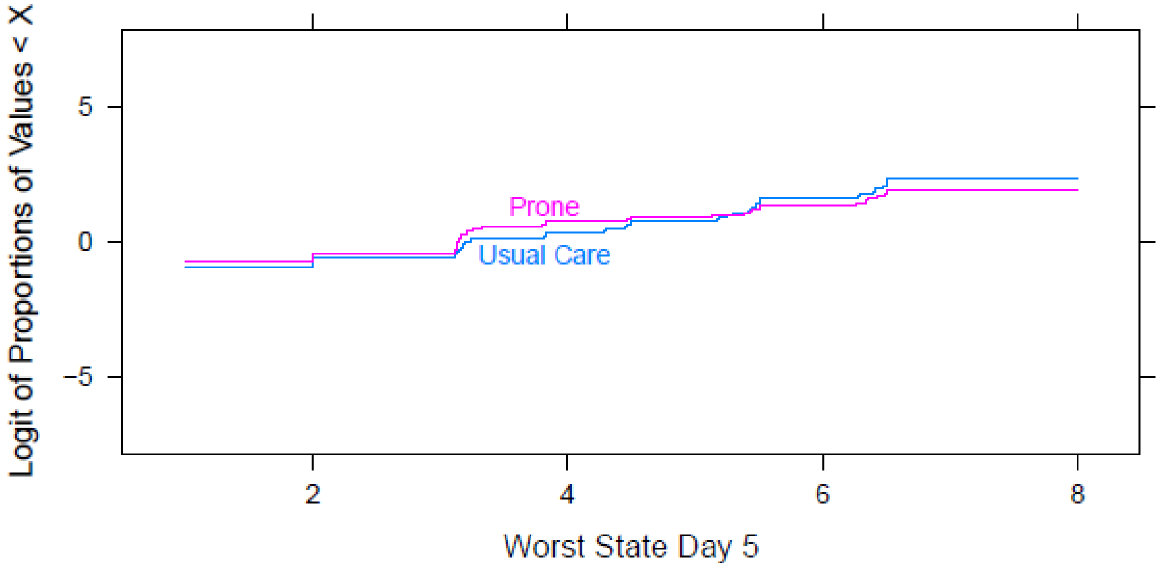


© 2022 Qian ET et al. *JAMA Internal Medicine*.

**eFigure 8.** Site by Treatment Interaction

Comparing model 1 (no interaction) to model 2 (includes treatment by site interaction)

```
## Method: stacking
## ------
##        weight
## model1 0.544
## model2 0.456
```

The model without the treatment by site interaction term has a 54% chance of being the correct model, over the model with the interaction term.

**Site**

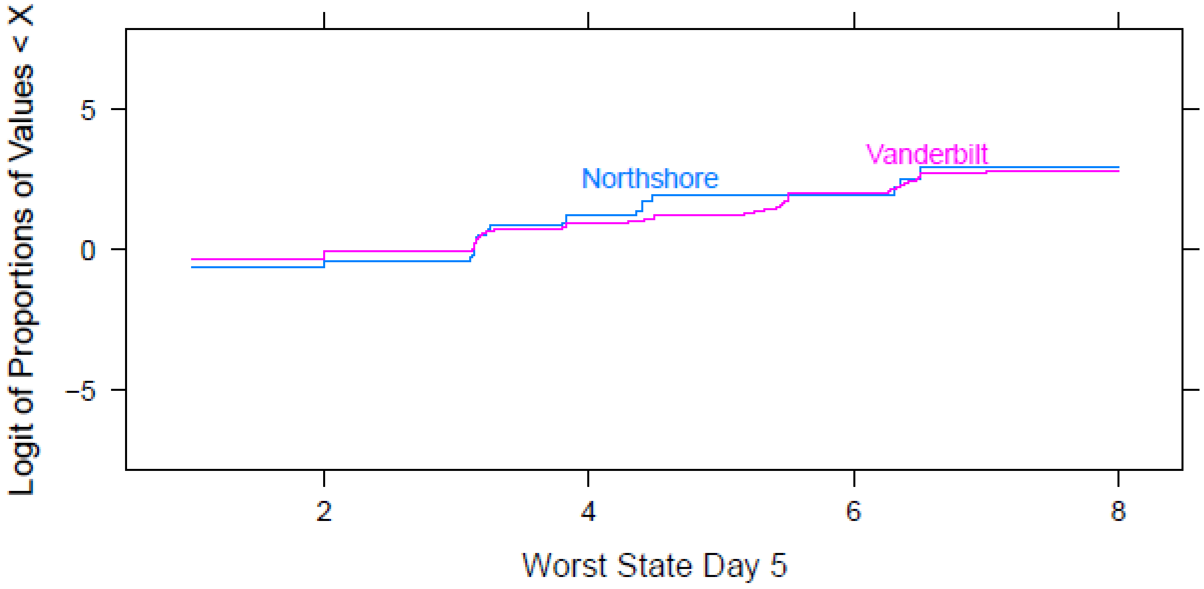


© 2022 Qian ET et al. *JAMA Internal Medicine*.

**Site: Vanderbilt**

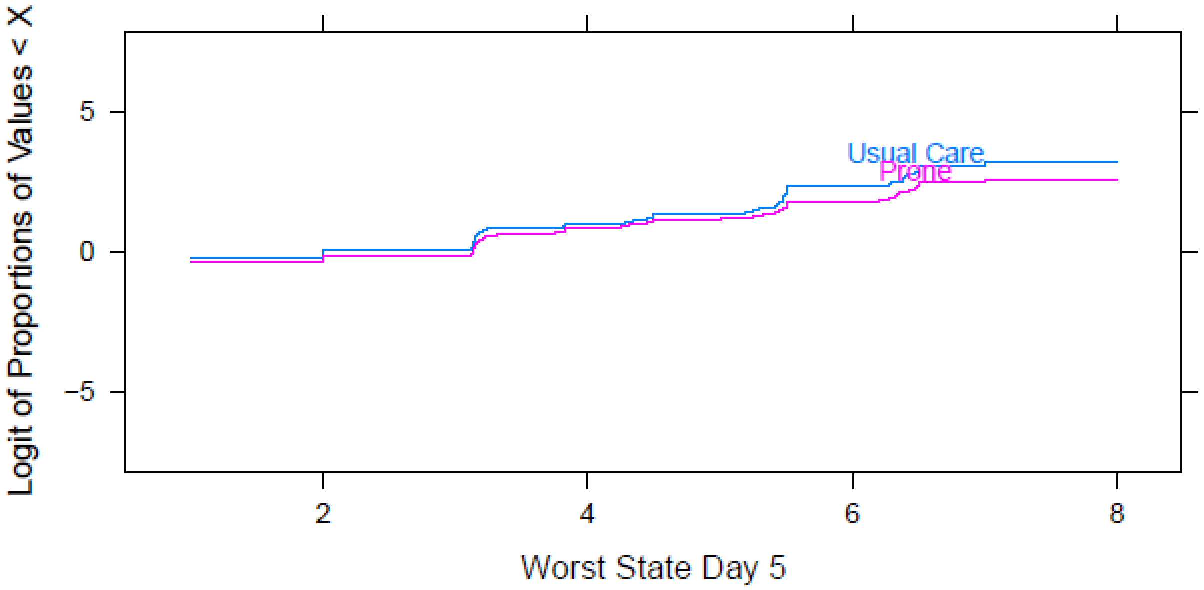


**Site: Northshore**

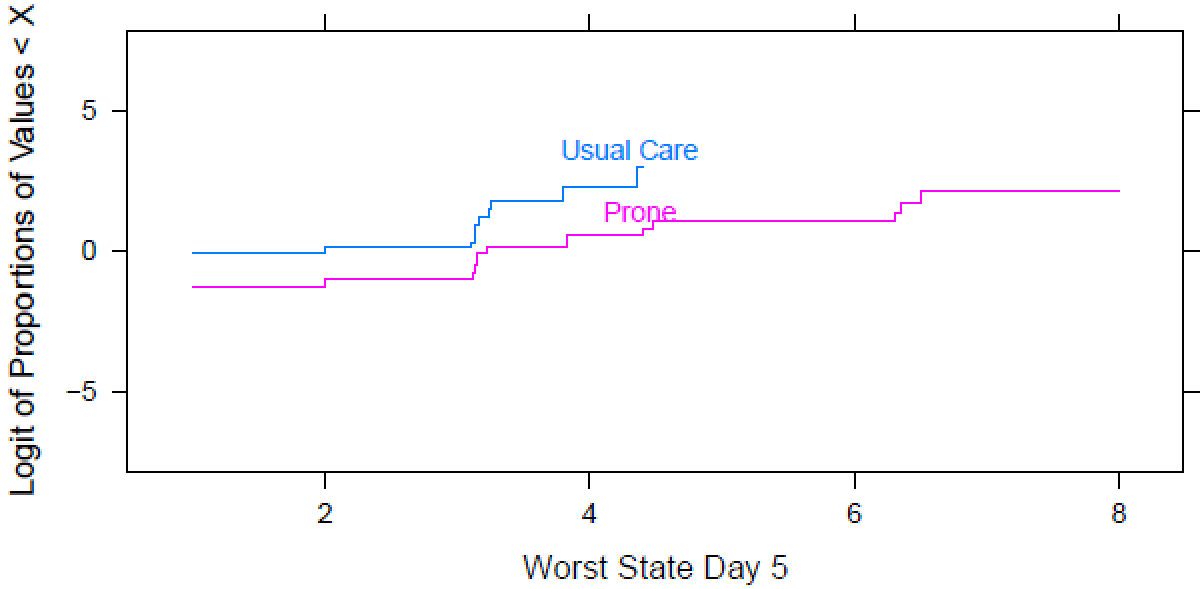


© 2022 Qian ET et al. *JAMA Internal Medicine*.
