# Supplementary material for: Assessment of Awake Prone Positioning in Hospitalized Adults With COVID-19: A Nonrandomized Controlled Trial
Source: JAMA Intern Med. 2022 Apr 18;182(6):612–21. doi: 10.1001/jamainternmed.2022.1070 (PMC9016608; doi:10.1001/jamainternmed.2022.1070)
Supplement: Supplement 3. — Nonauthor Collaborators [file jamainternmed-e221070-s003.pdf]

Supplemental Online Content: Nonauthor Collaborators

*Indicates required information. Only first name, last name, and suffix will appear in PubMed.

***Group Name(s):** Vanderbilt Learning Healthcare System Platform Investigators							
***First Name and Middle Initial(s)**	***Last Name**	***Suffix (eg, Jr, III)**	Academic Degrees	Institution	Location (city, state/province, country)	Role or Contribution, eg, chair, principal investigator	Group (if more than 1 Group listed in the byline) and/or Subgroup (eg, Steering Committee)
Robert	Dittus						
Shon	Dwyer						
Paul	Harris						
Tina	Hartert						
Jim	Hayman						
Catherine	Ivory						
Kevin	Johnson						
Ruth	Kleinpell						
Lee Ann	Liska						
Patrick	Luther						
Jay	Morrison						
Thomas	Nantais						
Mariann	Piano						
Kris	Rhem						
Russell	Rothman						
Matt	Semler						
Robin	Steaban						
Philip	Walker						
Consuelo	Wilkins						
Adam	Wright						
Autumn	Zuckerman						
