# Supplementary material for: Assessment of Awake Prone Positioning in Hospitalized Adults With COVID-19: A Nonrandomized Controlled Trial
Source: JAMA Intern Med. 2022 Apr 18;182(6):612–21. doi: 10.1001/jamainternmed.2022.1070 (PMC9016608; doi:10.1001/jamainternmed.2022.1070)
Supplement: Supplement 4. — Data Sharing Statement [file jamainternmed-e221070-s004.pdf]

Data Sharing Statement

Qian. Assessment of Awake Prone Positioning in Hospitalized Adults With COVID-19. *JAMA Intern Med*. Published April 18, 2022. doi:10.1001/jamainternmed.2022.1070

Data
Data available: Yes
Data types: Deidentified participant data, Data dictionary, Other (please specify)
Additional Information: Statistical analysis plan and analytic code will also be shared. The data will become available 3 months following publication of outcomes and will remain available for at least 5 years.
How to access data: Data will be made available to researchers who provide a methodologically sound proposal that has been approved by the Vanderbilt Institutional Review Board and the study executive committee.
When available: With publication

Supporting Documents
Document types: None

Additional Information
Who can access the data: Data will be made available to researchers who provide a methodologically sound proposal that has been approved by the Vanderbilt Institutional Review Board and the study executive committee.
Types of analyses: Data will be made available to researchers who provide a methodologically sound proposal that has been approved by the Vanderbilt Institutional Review Board and the study executive committee.
Mechanisms of data availability: Data will be made available to researchers who provide a methodologically sound proposal that has been approved by the Vanderbilt Institutional Review Board and the study executive committee.
